# Evidence for an association of gut microbial Clostridia with brain functional connectivity and gastrointestinal sensorimotor function in patients with irritable bowel syndrome, based on tripartite network analysis

**DOI:** 10.1186/s40168-019-0656-z

**Published:** 2019-03-21

**Authors:** Jennifer S. Labus, Vadim Osadchiy, Elaine Y. Hsiao, Julien Tap, Muriel Derrien, Arpana Gupta, Kirsten Tillisch, Boris Le Nevé, Cecilia Grinsvall, Maria Ljungberg, Lena Öhman, Hans Törnblom, Magnus Simren, Emeran A. Mayer

**Affiliations:** 1 G. Oppenheimer Center for Neurobiology of Stress & Resilience, UCLA Vatche and Tamar Manoukian Division of Digestive Diseases, UCLA CHS 42-210, MC737818, 10833 Le Conte Avenue, Los Angeles, CA 90095-7378 USA; 2 UCLA Department of Integrative Biology and Physiology, Los Angeles, USA; 3 Danone Nutricia Research, Innovation, Science and Nutrition, Palaiseau, France; 4 Department of Internal Medicine and Clinical Nutrition, Institute of Medicine, Sahlgrenska Academy, University of Gothenburg, Gothenburg, Sweden; 5 Department of Radiation Physics, Institute of Clinical Sciences, Sahlgrenska Academy, University of Gothenburg, Gothenburg, Sweden; 6 Department of Immunology and Microbiology, Institute of Biomedicine, Sahlgrenska Academy, University of Gothenburg, Gothenburg, Sweden; 7 Center for Functional Gastrointestinal and Motility Disorders, University of North Carolina, Chapel Hill, NC USA

**Keywords:** Central nervous system, Brain–gut axis, Brain imaging, Microbiome, Bacteria

## Abstract

**Background and aims:**

Evidence from preclinical and clinical studies suggests that interactions among the brain, gut, and microbiota may affect the pathophysiology of irritable bowel syndrome (IBS). As disruptions in central and peripheral serotonergic signaling pathways have been found in patients with IBS, we explored the hypothesis that the abundance of serotonin-modulating microbes of the order Clostridiales is associated with functional connectivity of somatosensory brain regions and gastrointestinal (GI) sensorimotor function.

**Methods:**

We performed a prospective study of 65 patients with IBS and 21 healthy individuals (controls) recruited from 2011 through 2013 at a secondary/tertiary care outpatient clinic in Sweden. Study participants underwent functional brain imaging, rectal balloon distension, a nutrient and lactulose challenge test, and assessment of oroanal transit time within a month. They also submitted stool samples, which were analyzed by 16S ribosomal RNA gene sequencing. A tripartite network analysis based on graph theory was used to investigate the interactions among bacteria in the order Clostridiales, connectivity of brain regions in the somatosensory network, and GI sensorimotor function.

**Results:**

We found associations between GI sensorimotor function and gut microbes in stool samples from controls, but not in samples from IBS patients. The largest differences between controls and patients with IBS were observed in the *Lachnospiraceae incertae sedis*, Clostridium XIVa, and Coprococcus subnetworks. We found connectivity of subcortical (thalamus, caudate, and putamen) and cortical (primary and secondary somatosensory cortices) regions to be involved in mediating interactions among these networks.

**Conclusions:**

In a comparison of patients with IBS and controls, we observed disruptions in the interactions between the brain, gut, and gut microbial metabolites in patients with IBS—these involve mainly subcortical but also cortical regions of brain. These disruptions may contribute to altered perception of pain in patients with IBS and may be mediated by microbial modulation of the gut serotonergic system.

**Electronic supplementary material:**

The online version of this article (10.1186/s40168-019-0656-z) contains supplementary material, which is available to authorized users.

## Introduction

Irritable bowel syndrome (IBS) is a common disorder characterized by chronically recurring abdominal pain associated with altered bowel habits [1]. Even though alterations in the brain-gut-microbiome axis have been implicated as an important component, IBS pathophysiology and the role of altered gut microbiome-brain interactions are incompletely understood [1]. Previous brain imaging studies have identified functional and structural differences within the brain, including in regions of the sensorimotor, salience, and emotion regulating networks between healthy controls (HCs) and IBS patients [2, 3].

Visceral hypersensitivity and gastrointestinal (GI) motor function abnormalities are considered to be of primary importance for IBS symptom generation. Balloon distension in different parts of the GI tract, during which patients report the extent of pain and discomfort experienced, has been used to assess enhanced perception of visceral stimuli (“evoked visceral hypersensitivity”) in patients with functional GI disorders. A quantitative meta-analysis focused on changes in brain activation during rectal distension identified greater engagement of regions associated with endogenous pain processing and modulation, such as the basal ganglia, in IBS compared to HCs [4]. Studies involving large cohorts of IBS subjects have shown modest associations between GI symptom severity and evoked visceral sensitivity, assessed through balloon distension [5, 6], as well as between IBS symptoms and abnormal GI motor function [7]. These modest associations may reflect a role for other aspects of brain-gut signaling, that may play a similar or greater role in symptom generation.

Reports of alterations in the gut microbiome in patients meeting IBS symptom criteria have been inconsistent, likely due to heterogeneity of gut microbial composition in IBS, differences in sampling and analysis protocols, and a lack of information about the causality of brain gut microbiome interactions in symptom generation [8, 9]. Despite these inconsistent findings, several pieces of evidence suggest a role of Clostridiales-associated species in IBS. In a previous study, we have demonstrated that enterotypes composed of Clostridiales-associated species comprise a multivariate microbial signature that associates with IBS symptom severity [10]. In another study, we showed that the gut microbial composition of IBS patients shows an increase in members of the class Clostridia, which correlates with volumes of brain regions in the sensorimotor network [11]. Importantly, recent evidence obtained in mouse models reveals an important role for colonic spore-forming bacteria in the order Clostridiales (particularly enriched with members of families *Ruminococcaceae* and family *Lachnospiraceae*) in stimulating the biosynthesis and release of serotonin (5-HT) from intestinal enterochromaffin cells and modulating GI motility [12]. Dysfunctions of the serotonergic system have been extensively studied within the context of IBS due to the prominent role of 5-HT in secretion, absorption, and intestinal transit in the GI tract, and mood, pain modulation, and cognitive function within the central nervous system (CNS) [13]. Therapeutic agents targeting 5-HT signaling, including direct 5-HT receptor modulators and 5-HT selective reuptake inhibitors, have been investigated as treatment options, achieving moderate efficacy in certain patients with IBS [14]. Additionally, several studies have demonstrated correlations between 5-HT plasma levels and post-prandial symptomology and sigmoid colon activity [13, 15, 16].

Although the mechanisms driving gut microbiota-initiated signaling to the brain in humans are not known, this interaction may be mediated by the microbial production of small-molecules, which can signal to the brain via afferent vagal fibers, the immune system, or directly through the circulation. Examples include short-chain fatty acids or secondary bile acids, which have been documented to interact with specific receptors on enterochromaffin cells, thereby modulating the gut serotonergic system [12]. Based on the close physical connections between enterochromaffin cells and vagal afferent nerve terminals, such modulation could lead to 5-HT-mediated vagal afferent signaling to the medullary nucleus tractus solitarius and to higher emotional and autonomic networks in the brain. The vagus nerve is a key component in the microbiota-gut-brain axis interface [17] and a major pathway providing the brain with interoceptive as well as microbial information from the gut. Further support for microbial generated metabolites in IBS symptom generation include the beneficial effects of diets that eliminate fermentable oligosaccharides, disaccharides, monosaccharides, and polyols (FODMAP) [18]. FODMAPs are short-chain carbohydrates that are poorly absorbed and rapidly fermented by gut bacteria to produce metabolites that can influence the gut microbiota, gut barrier, immune response, and visceral sensation [19]. In addition to their role in gut brain signaling, microbial-derived metabolites may directly activate the enteric nervous system, modulating contractile activity and secretion [20].

In addition to identifying functional alterations in specific brain regions, more recent efforts have focused on identifying alterations in the architecture and connectivity of brain networks [21]. Brain connectivity can be assessed using structural and functional network analysis via graph theory. Within this framework, brain regions are characterized by measures that quantify their contribution to the functional and anatomical integrity and information flow in the whole brain network [22–25].

In this exploratory study, we build on the considerable evidence implicating certain gut microbes (order *Clostridiales*) and regions of the sensorimotor network in IBS. We use a biological system-based, data-driven approach to interrogate and visualize the relationship between relative abundance of several species in the order Clostridiales (families *Ruminococcaceae* and *Lachnospiraceae*) with GI sensorimotor function and connectivity of key regions of the brain’s sensorimotor network in HC and IBS patients. Even though findings from this exploratory study will need to be validated in a much larger sample, they provide initial support for role of alterations in the brain-gut-microbiome axis in symptom generation in IBS.

## Methods

### Subjects

Adult IBS patients (*n* = 65), aged 18–65 years, were recruited between 2011 and 2013 at a secondary/tertiary care outpatient clinic (Sahlgrenska University Hospital, Gothenburg, Sweden). As subjects were recruited in 2011–2013, IBS was diagnosed based on the Rome III criteria [26]. A gastroenterologist (H.T. or M.S.) diagnosed the patients. Exclusion criteria included (1) the use of probiotics or antibiotics during the study period or within 1 month before inclusion; (2) another diagnosis that could explain the GI symptoms such as celiac disease, inflammatory bowel disease, or microscopic colitis; (3) severe psychiatric disease as the dominant clinical problem; (4) any other severe diseases; and (5) a history of drug or alcohol abuse. This study did not focus on only one subgroup of IBS as previous studies [10, 11, 27] have shown only modest associations between IBS subtype defined by symptom criteria, gut microbiota composition, and brain imaging differences. A HC group (*n* = 21) was recruited by use of advertisement and checked by interview and questionnaire to exclude chronic diseases and any current GI symptoms. All subjects were of Caucasian origin. All subjects completed a questionnaire to characterize their symptom severity using the IBS Severity Scoring system (IBS-SSS), and according to validated cut-off levels, the patients were categorized as having mild IBS (score of < 175), moderate IBS (175–300), or severe IBS (> 300) [28]. Even though the IBS-SSS has not been validated in healthy subjects, we use it here as a proxy for the assessment of mild abdominal symptoms, not meeting IBS diagnostic criteria. Data from these subjects has been previously published as a part of a larger study [10]. Here, we analyze a subset of individuals, 65 IBS patients (46 females) and 21 HCs (16 females), all of whom underwent functional brain imaging. Among HCs, none of the subjects were taking any medications. Among IBS patients, seven subjects (11%) were taking a proton-pump inhibitor and ten subjects were taking a daily psychotropic medication. As mentioned above, these medications were discontinued 48 h prior to investigation.

All investigations were performed during a 1-month period, and in the same order for each patient with oroanal transit time (OATT) and rectal sensitivity testing performed during the same day, MRI 7–10 days after that, stool sample collection 3–5 days thereafter, and the lactulose challenge test at the end of this month.

All subjects were given verbal and written information about the study prior to providing written informed consent to participate. The Regional Ethical Review Board in Gothenburg approved the study before the start of subject inclusion.

### Measurement of GI sensorimotor function

#### Evoked visceral sensitivity: rectal barostat procedures

After an overnight fast and a 500–800 mL tap water enema, a balloon catheter was inserted into the rectum, so that the middle of the bag was located approximately 10 cm from the anal verge, and connected to a computer-driven electronic barostat (Dual Drive Barostat, Distender Series II; G&J Electronics Inc., Toronto, ON, Canada). After a 20–30-min recovery period, the pressure in the balloon was increased from 4 mmHg in steps of 1 mmHg for 1 min per step until respiratory excursions were observed. The baseline operating pressure (BOP) was defined as 2 mmHg above the minimal distension pressure at which respiratory excursions were clearly recorded from the barostat tracing. Next, an initial conditioning distension sequence was performed in which the pressure was increased from 0 to 20 mmHg in steps of 4 mmHg for 15 s per step. Thereafter, an ascending method of limits rectal distension protocol was used, with ramp inflation starting at 0 mmHg increasing by steps of 4 mmHg with 1 min per step to identify sensory thresholds. For this study, we used *rectal pain and discomfort thresholds*, in addition to *first rectal sensation*. The distension sequence progressed until pain was reported or until the maximal distension of 60 mmHg was achieved [29].

During the second half of the procedure, subjects randomly received fixed phasic distensions at 12, 24, 36, and 48 mmHg above the BOP and were asked to complete visual analogue scale ratings for rectal sensations after 30 s of distension. The distension lasted for 60 s with an inter-stimulus interval of 2 min. During the phasic distension, only one distension level above the pain threshold was delivered for ethical reasons. Most participants completed the 12 and 24 mmHg distension levels, but fewer continued to 36 and 48 mmHg distension. For this reason, we included visual analogue scale ratings for the 24 mmHg phasic distension in our analyses, and only pain and discomfort rating were used in this study (*rectal pain and discomfort intensity,* respectively).

#### OATT

OATT using a radiopaque marker study [7] was determined in all subjects. Any medications with known effects on the GI tract (proton pump inhibitors, laxatives, antidiarrheals, opioid analgesics, prokinetics, spasmolytics, antidepressants) were discontinued at least 48 h before intake of the first radiopaque markers. The subjects ingested ten radiopaque markers each day for 6 days. On the morning of the 7th day, the radiopaque rings still present in the bowel were counted upon arrival at the laboratory, using fluoroscopy (Exposcop 7000 Compact, Ziehm GmbH, Nüremberg, Germany). *OATT* expressed in days was calculated by dividing the number of retained radiopaque markers by the daily dose number; i.e., 10.

#### Nutrient and lactulose challenge test

This challenge has been previously described in detail [30]. Briefly, all participants arrived to the laboratory at 7:30 AM after an overnight fast. The combined nutrient and lactulose challenge test (400 mL Nutridrink (Danone Nutricia, Utrecht, The Netherlands), 1.5 kcal/mL, 16% protein, 49% carbohydrate, 35% fat, gluten free, lactose < 0.025 g/100 mL, 25 g lactulose) was served at 8:00 AM. The severity of 8 GI symptoms and the overall digestive comfort were assessed every 15 min starting 30 min before the test meal and during 4 h after meal intake. In this study, we only included the 4 h mean area under the curves for the pain and discomfort ratings during the challenge test (*lactulose challenge*—*pain and discomfort*, respectively).

### Fecal sample DNA extraction and microbial composition assessment

Fecal samples were collected in RNAlater solution (Ambion, Courtaboeuf, France) at subjects’ homes, stored at room temperature, and were returned to the laboratory within 3–4 days in the majority of cases. In a small number of cases, samples were returned within 3 weeks. DNA was extracted using mechanical lysis (Fastprep FP120; ThermoSavant, Illkirch, France) followed by phenol/chloroform-based extraction as previously described [31]. Hypervariable 16S ribosomal RNA (rRNA) regions (V5–V6) were amplified using primers 5′-AGGATTAGATACCCTGGTA-3′ and 5′-CRRCACGAGCTGACGAC-3′. DNA Vision SA (Charleroi, Belgium) performed sequencing using a 454 Life Sciences Genome Sequencer FLX instrument (Roche, Basel, Switzerland). Raw reads quality filtering and trimming, operational taxonomic units (OTU) clustering, and taxonomic assignment were performed using the LotuS v1.32 pipeline [32] as previously described [10].

### Resting state neuroimaging

T1-weighted images were acquired on a 3 Tesla Philips Achieva using the standard 8-channel head coil, with a T1-weighted 3D TFE gradient echo scan, i.e., a magnetization-prepared rapid acquisition gradient echo (MP-RAGE) sequence, repetition time = 7.0 ms, echo time = 3 .2 s, inversion time = 900 ms, flip angle = 9°, field of view = 256 by 220 mm, slices per volume = 176, slice thickness = 1 mm, voxel size = 1 × 1 × 1 mm. Resting state fMRI (eyes closed) was acquired using Philips SENSE parallel imaging with a reduction factor = 2, repetition time = 2000 ms, echo time = 30 ms, flip angle = 77°, field of view 250 × 250 mm, acquisition matrix = 72 × 72, slice thickness = 4 mm, reconstructed voxel size = 4 × 2.6 × 2.6 mm. In preparations before the scan, the subjects were asked not to take any analgesics, anti-histamines, alcohol, or nicotine for a minimal of 12 h before the scan; not to take any psychopharmacological drugs for 48 h before the scan; not to ingest caffeine for 2–3 h before the scan; and to avoid strenuous physical activity on the day before the scan.

#### Quality control

Preprocessing for quality control involved bias field correction, coregistration, motion correction, spatial normalization, tissue segmentation, and Fourier transformation for frequency distribution were performed at the Bioinformatics and Neuroimaging core of the G. Oppenheimer Center for Neurobiology of Stress and Resilience at UCLA. Functional images were included based on compliance with acquisition protocol, full brain coverage, motion estimate of < ½ voxel size between adjacent time points, low standard deviation across time series for all voxels, ghosting in cerebrum, minimal physiological noise (> 0.2 Hz in frequency spectrum), and few to no outlier voxels, mean intensity shifts, or K-space “spikes.”

#### Brain Parcellation

Segmentation and regional parcellation of gray matter images was performed using FreeSurfer (Dale, Fischl et al. 1999; Fischl, Sereno et al. 1999; Fischl, Salat et al. 2002) and workflow pipelines at UCLA developed in collaboration with the UCLA Laboratory of Neuroimaging pipeline using the Destrieux atlas and the Harvard-Oxford atlas [24]. This parcellation yielded 74 cortical structures, 7 subcortical structures, the cerebellum, and the brainstem, for a complete set of 165 parcellations of the entire brain.

#### Functional brain network construction

The parcellation and the functional connectivity results were combined to produce a 165 × 165 weighted, undirected connectivity matrix. Resting-state image preprocessing was performed using the SPM8 software (Wellcome Department of Cognitive Neurology, London, UK). Images were transformed from DICOM into NIfTI, slice-time corrected, co-registered with the high-resolution structural images, spatially normalized to the MNI space, and resampled to a voxel size of 2 × 2 × 2 mm. Normalized functional images were further preprocessed and analyzed using the SPM-based CONN toolbox version 13 (www.nitrc.org/projects/conn). The resting-state images were filtered using a band-pass filter (0.008/s < *f* < 0.08/s) to reduce the low- and high-frequency noises. A component based noise-correction method, CompCor [33], was applied to remove nuisances for better sensitivity and specificity of the analysis. Six motion realignment parameters and confounds for white matter and CSF were removed using regression. Region-to-region functional connectivity, cross-correlations between the blood oxygenated level-dependent time series, were computed between all the brain regions in CONN toolbox. The connectivity correlation coefficients were then used to construct the final functional network setting negative values to zero. The magnitude of the correlation represents the weights of the specific region in the functional network.

#### Computing network metrics

The Graph Theoretic GLM tool (www.nitrc.org/projects/metalab_gtg) [34] and in-house matlab workflow scripts were used to calculate and analyze the functional brain network properties and organization from the subject-specific functional brain networks. Several regional weighted network metrics indexing centrality were computed. Regions with high centrality are highly influential within a network, communicate with many other regions, facilitate functional integration, and play a key role in network resilience to insult [25]. Three indices of centrality were computed: (1) *Degree strength* reflects the number of other regions a brain region interacts with functionally (local prominence), (2) *betweenness centrality*, reflecting the ability of a region to influence information flow (signaling) between two other regions, and (3) *eigenvector centrality*, where higher values indicate the region is directly connected to other highly connected regions reflective of the global (vs. local) prominence of a region.

### Selection of microbial genera

In order to increase statistical power, we selected genera based on previous work, which showed that spore formers particularly enriched with members of order Clostridiales, families *Ruminococcaceae* and *Lachnospiraceae*, stimulate the biosynthesis and release of 5-HT from intestinal enterochromaffin cells and modulate GI motility [11, 12]. Only genera in these families were included in our analysis. Any genus with greater than 80% missing data in either the IBS or HC group was excluded. The genera that fulfilled these criteria and were included in this study are families *Ruminococcaceae* (genera: *Clostridium IV*, *Faecalibacterium*, and *Oscillibacter*) and *Lachnospiraceae* (genera: *Clostridium XIVa*, *Clostridium XIVb*, *Blautia*, *Coprococcus*, *Roseburia*, and *Lachnospiracea incertae sedis*).

### Statistical analysis

Tripartite network analysis was performed to integrate information from three data sets: (1) abundance of bacterial genera (relative to all other genera) in the order Clostridiales including families *Ruminococcaceae* and *Lachnospiraceae*, (2) GI sensorimotor function data, including IBS-SSS, and (3) the functional network metrics characterizing regions of interest (Table 1), including mid- and posterior insula (m and pINS), somatosensory cortex, basal ganglia (nucleus accumbens [NAcc], caudate nucleus, putamen, and pallidum), and thalamus. For reasons similar to the restriction of microbial taxa, the selection of brain regions of a sensorimotor network was based on previous structural and resting state brain imaging studies in IBS consistently demonstrating alterations in the regions of the sensorimotor network, with a recent study showing associations between gut microbial abundance and differences in sensorimotor regions [11, 35–37] (Additional file 1: Table S1 and Additional file 2: Table S2).

**Table 1:** Regions of Interest from the Destrieux and Harvard-Oxford Atlas

Region	Description	Abbreviation
Primary somatosensory cortex (S1)	Postcentral gyrus	PosCG
	Postcentral sulcus	PosCS
	Central sulcus	CS
Secondary somatosensory cortex (S2)	Subcentral gyrus (central operculum) and sulcus	SbCG_S
Primary motor cortex (M1)	Precentral gyrus	PRCG
	Inferior part of the precentral sulcus	InfPrCS
	Superior part of the precentral sulcus	SupPrCs
Supplementary motor area (M2/SMA)	Superior frontal gyrus	SupFG
	Superior frontal sulcus	SupFS
Anterior/middle insula (a/mINS)	Superior segment of the circular sulcus of the insula	SupCirInS
Middle/posterior insula (m/pINS)	Long insular gyrus and central sulcus of the insula	LoInG_CInS
	Inferior segment of the circular sulcus of the insula	InfCirInS
Posterior insula (pINS)	Posterior ramus of the lateral sulcus	PosLS
Basal ganglia	Putamen	Pu
	Caudate nucleus	CaN
	Nucleus accumbens	NAcc
	Globus pallidus	Pal
Thalamus	Thalamus	Tha

The interaction between the phenome (GI sensorimotor function and IBS symptoms), microbiome (stool microbial community), and connectome (indices of regional centrality in the brain) was determined by computing Spearman’s correlations between different data types controlling for age and sex in Matlab version R2015b. An effect size of *r* = 0.50 is considered large (25% variance explained), 0.30 medium (9% variance explained), and 0.10 small (1% variance explained) [38]. Fisher’s r-to-z transformation was applied to evaluate the correlation coefficient difference between groups (IBS-HC), using the *Z* test. Significance was considered at uncorrected *p* < 0.05. Parameter estimates and significance values from the group comparison are provided in Additional file 3: Table S3. No corrections for multiple testing were employed as our goal for this exploratory research was to generate a number of hypotheses for further testing and confirmation in a larger sample. Using G*Power, we perform a post-hoc analysis to determine the effect size, *r*, our samples could detect with adequate power (80%) based on an alpha = 0.05 (uncorrected *p*), and a two-tailed test for a significant correlation. For the IBS sample (*N* = 65), we only had adequate power to detect a significant correlation of *r* ≥ 0.33, if it existed. For the HC sample (*N* = 21), we only had adequate power for *r* ≥ 0.54. We provide further details regarding cohort sizes needed to justify tighter error control in Additional file 4.

To visualize and construct brain, GI sensorimotor function, and stool microbial community interaction networks, Cytoscape v. 3.5.1 was used. First, the difference network (and subnetworks), representing group differences at *p* < 0.05, were constructed using the unweighted force-directed layout. This layout uses a physics algorithm to simulate the network as a physical system, where edges attract and nodes repel. This technique organizes the network to avoid crossing edges and overlapping nodes. As such, nodes that are connected and have similar associations are grouped together, allowing for clusters or patterns in the data to emerge. Associations for this network were included if the Pearson’s correlations for the IBS and/or the HCs data achieved significance. For genera-specific subnetworks, we select the genus of interest and its first neighbors as nodes and all adjacent edges. The architecture of IBS and HCs networks and subnetworks were based on the significant difference network. Edges (lines) represent significant *Z* values from the group test and nodes represent data points. Positive *Z* values were colored red and indicate IBS > HC and a negative *Z* value indicates IBS < HC. Individual edge estimates by group are reported in Additional file 1: Table S1 and Additional file 2: Table S2. The results are described in terms of direct (correlations with genera) and indirect effects (GI sensorimotor function and brain connectivity metric that are a part of the interaction network, but not directly correlated with the genus of interest) with a focus on the interaction between genus and GI sensorimotor function. As all subnetworks are derived from significant data identified in the difference network, the analysis presumes that associations present in one group that are missing in another (1) differentiate the groups, and (2) indicate potential clues to the functionality of the system, permitting hypothesis generation for future research. Independent sample *t* tests were used to test for group differences in clinical variables.

## Results

### Clinical and behavioral characteristics

Means, standard deviations, and group comparisons for age, symptom severity, and GI sensorimotor function variables are presented in Table 2**.** No differences in age were noted. As expected, IBS subjects reported significantly higher discomfort and pain ratings during the 24 mmHg rectal balloon distension and during the nutrient and lactulose challenge test. In contrast, no statistically significant differences between the groups were observed for rectal discomfort or pain thresholds, and OATT. On the IBS-SSS, 12 patients were classified as mild, 24 moderate, and 29 severe symptoms. All HCs were classified as mild/no symptoms.

**Table 2:** Subject characteristics and GI sensorimotor function measures

	Healthy controls (*N* = 21, *F* = 16)		IBS (*N* = 65, *F* = 46)		*t*	*p*
	M	SD	M	SD		
Age	33.3	9.56	33.3	10.2	0.004	1.00
Rectal discomfort threshold (mmHg)	24.4	7.1	21.2	7.8	1.66	0.10
Rectal pain threshold (mmHg)	30.5	10.3	27.4	8.8	1.33	0.19
First rectal sensation (mmHg)	10.3	5.1	9.9	5.9	0.23	0.82
IBS-SSS	26	31.3	280	100	− 11.36	1.15 × 10^−18^
Rectal discomfort intensity (VAS, mm)	49.8	22.1	68.7	25.7	− 3.05	0.003
Rectal pain intensity (VAS, mm)	25.4	31.33	40.4	31.5	− 2.4	0.019
Lactulose challenge—pain (AUC)	60.7	166.41	1139.4	1243.07	− 3.95	0.0002
Lactulose challenge—discomfort (AUC)	212.5	227.9	1911.1	1062.2	− 7.23	1.91 × 10^− 10^
OATT (days)	1.5	0.7	1.6	1.2	− 0.26	0.84

### Tripartite network analysis of gut microbe-brain connectivity

**Fig. 1:**
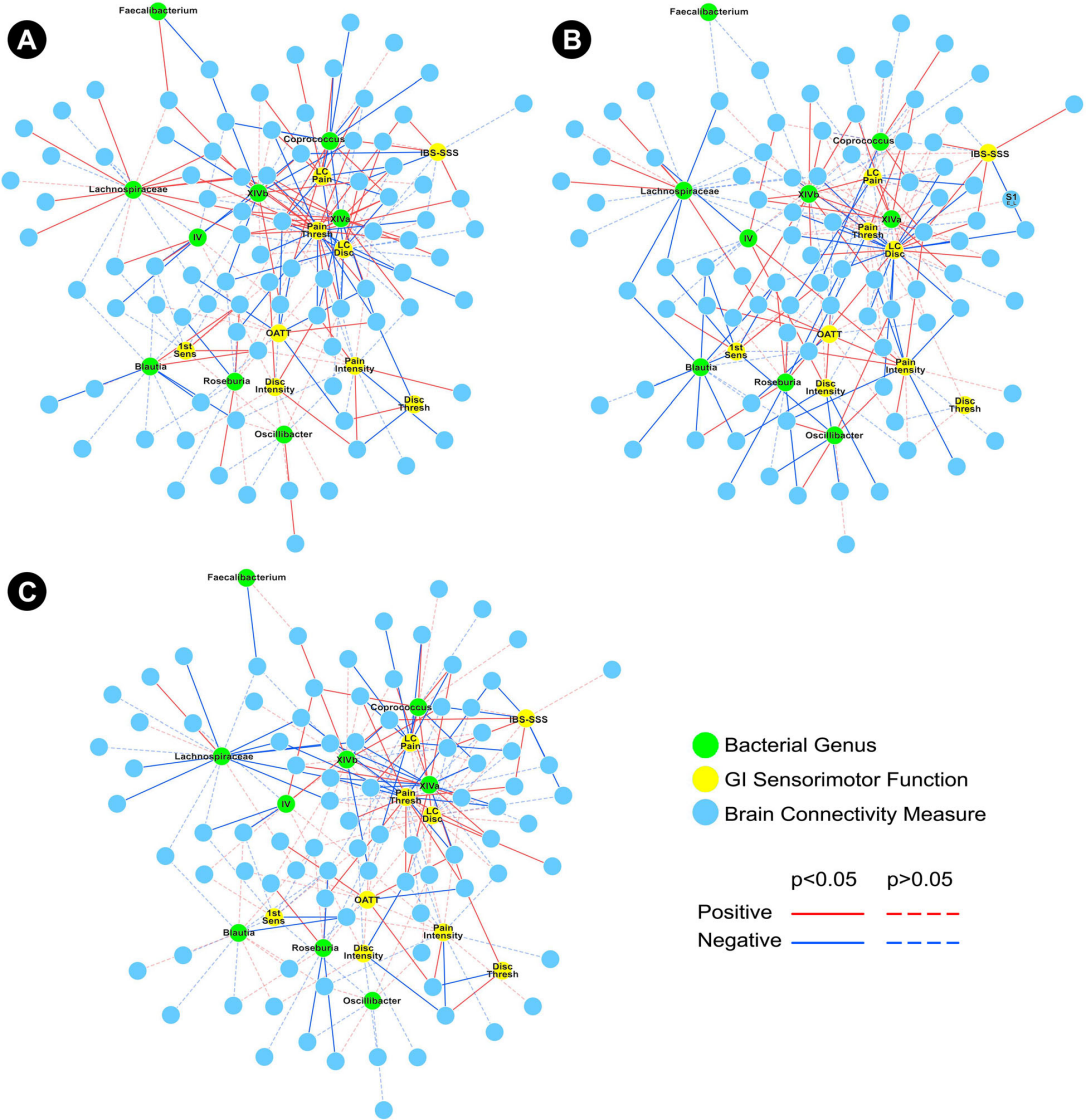
Global tripartite microbial genus-brain connectivity-evoked symptom network. **a** Demonstrates the global tripartite healthy controls network. **b** Demonstrates the global tripartite IBS network. **c** Demonstrates the global tripartite difference network. For clarity, labels related to brain connectivity measures have been omitted from these global networks. *1st Sens* first sensation threshold during balloon distension, *Disc Thresh* discomfort threshold during balloon distension, *IBS*-*SS* irritable bowel syndrome–scoring system scores, *IV Clostridium IV*, *LC Disc* discomfort during lactulose challenge test, *LC Pain* pain during lactulose challenge test, *OATT* oroanal transit time, *Pain Thresh* pain threshold during balloon distension, *Disc Intensity* visual analogue scale rating of discomfort during 24 mmHg distension, *Pain Intensity* visual analogue scale rating of pain during 24 mmHg distension, *IBS*-*SSS* IBS Severity Scoring System, *XIVa Clostridium XIVa*, *XIVb Clostridium XIVb*, *Lachnospiraceae Lachnospiraceae incertae sedis*

The global tripartite microbial-brain-evoked symptom networks are depicted in Fig. 1a (HC), b (IBS), and c (difference network). These networks show statistical associations between brain, microbial, and GI sensorimotor function parameters. Additional file 1: Table S1, Additional file 2: Table S2, and Additional file 3: Table S3 outline all of the associations for HCs, IBS, and difference networks, respectively, in greater detail. In the following, we describe the results with respect to microbial subnetworks related to four genera within the order Clostridiales: *L*. *incertae sedis, Coprococcus*, and *Clostridium XIV*a and b.

#### *Lachnospiracea incertae sedis*

**Fig. 2:**
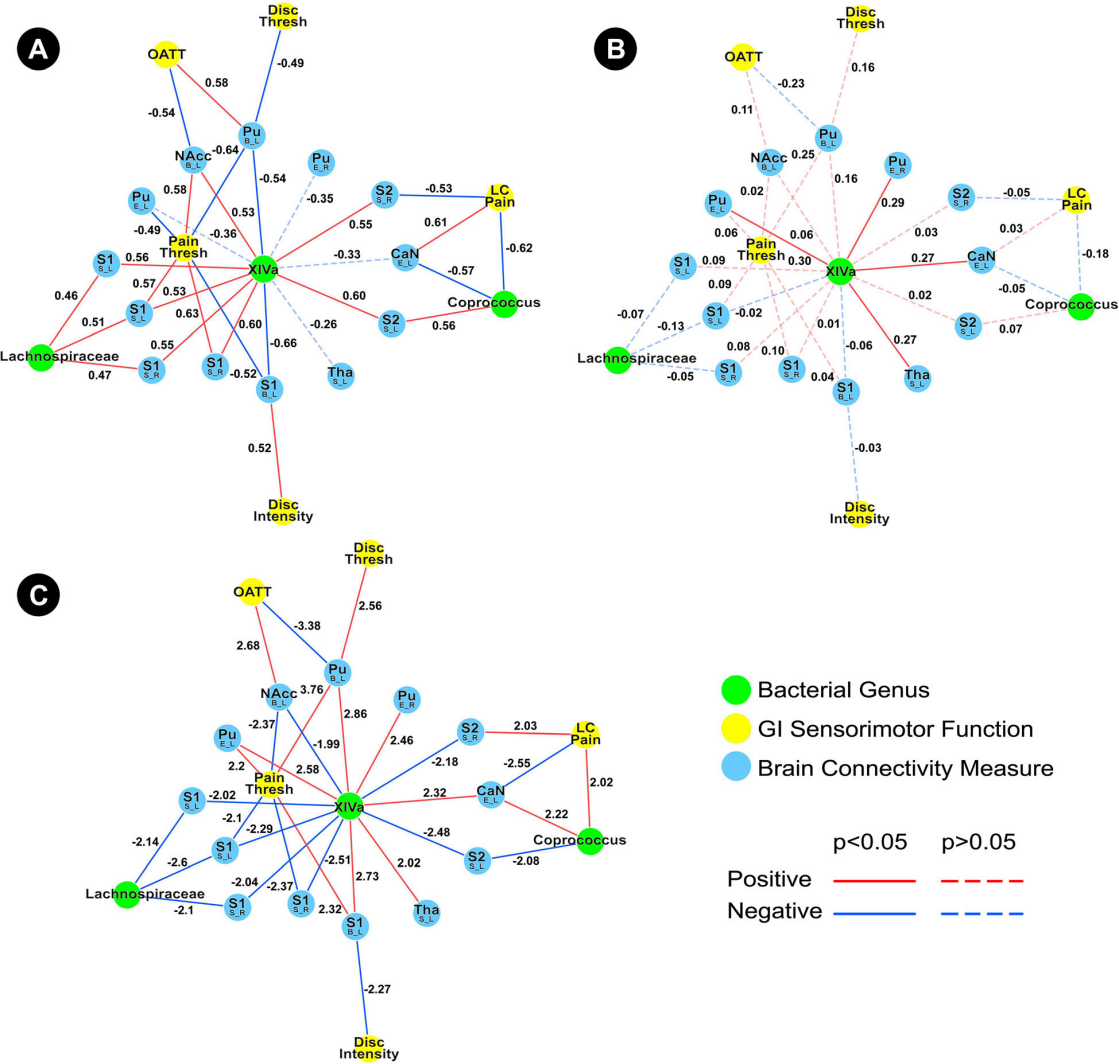
Tripartite *Lachnospiraceae incertae sedis* subnetwork. **a** Demonstrates the *Lachnospiracea incertae sedis* healthy controls subnetwork. **b** Demonstrates the *Lachnospiracea incertae sedis* IBS subnetwork. **c** Demonstrates the *Lachnospiracea incertae sedis* difference network. Functional brain connectivity of regions of interest is presented with the region of interest noted in a larger font, with the connectivity measure and lateralization indicated below in the form X_Y, where *X* indicates a connectivity measure (*B* betweenness centrality, *E* eigenvector centrality, *S* degree strength) and *Y* indicates lateralization (*L* left, *R* right). *Pain Thresh* pain threshold during balloon distension, *XIVa Clostridium XIVa*, *Lachnospiraceae Lachnospiraceae incertae sedis*

The HCs subnetwork showed a robust positive association network between *Lachnospiraceae incertae sedis* and S1, in addition to an indirect association between this genus with rectal pain threshold through connectivity of S1 (Fig. 2a). In contrast to the HC association network, the IBS subnetwork demonstrated *no* associations between *Lachnospiraceae incertae sedis* and pain threshold, but did show a single positive association between this genus and connectivity of S2 (Fig. 2b). The *Lachnospiraceae incertae sedis* difference network underscores that the observed differences are statistically significant (Fig. 2c). *Clostridium XIVa* was also a part of the *Lachnospiraceae incertae sedis* subnetwork.

#### Coprococcus

**Fig. 3:**
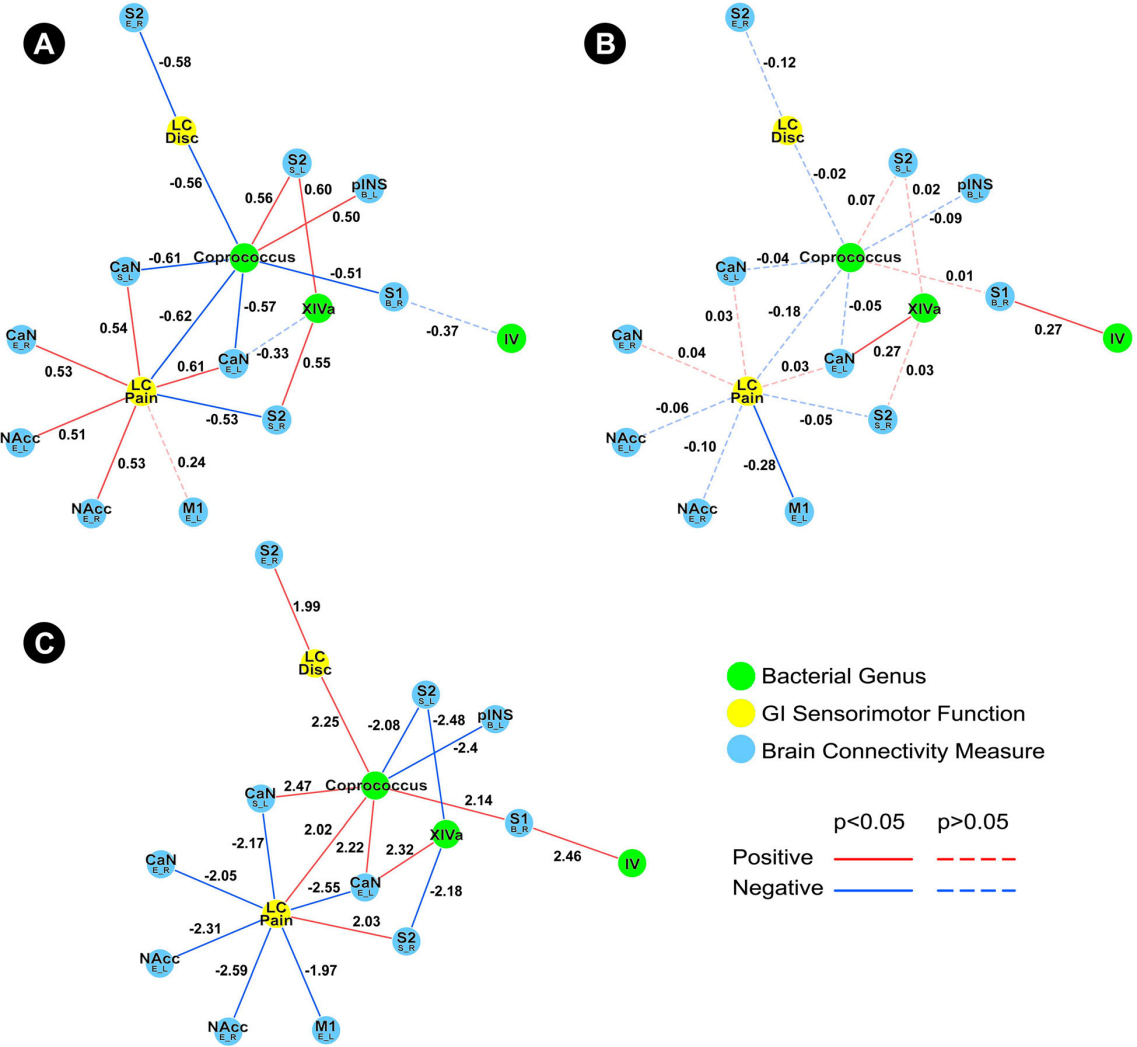
Tripartite *Coprococcus* Subnetwork. **a** Demonstrates the *Coprococcus* healthy controls subnetwork. **b** Demonstrates the *Coprococcus* IBS subnetwork. **c** Demonstrates the *Coprococcus* difference network. Functional brain connectivity of regions of interest is presented with the region of interest noted in a larger font, with the connectivity measure and lateralization indicated below in the form X_Y, where *X* indicates a connectivity measure (*B* betweenness centrality, *E* eigenvector centrality, *S* degree strength) and *Y* indicates lateralization (*L* left, *R* right). *IV Clostridium IV*, *LC Disc* discomfort during lactulose challenge test, *LC Pain* pain during nutrient and lactulose challenge test, *XIVa Clostridium XIVa*

The HC subnetwork showed direct negative associations between *Coprococcus* and discomfort and pain during the nutrient and lactulose challenge test, in addition to indirect association between this genus and pain reported during this challenge test through connectivity of the caudate (Fig. 3a). In contrast, the IBS subnetwork did not show any significant associations involving *Coprococcus* (Fig. 3b). The *Coprococcus* difference network underscores that the observed differences are statistically significant (Fig. 3c). *Clostridium XIVa* and *Clostridium IV* were also a part of the *Coprococcus* subnetwork.

#### Clostridium XIVa

**Fig. 4:**
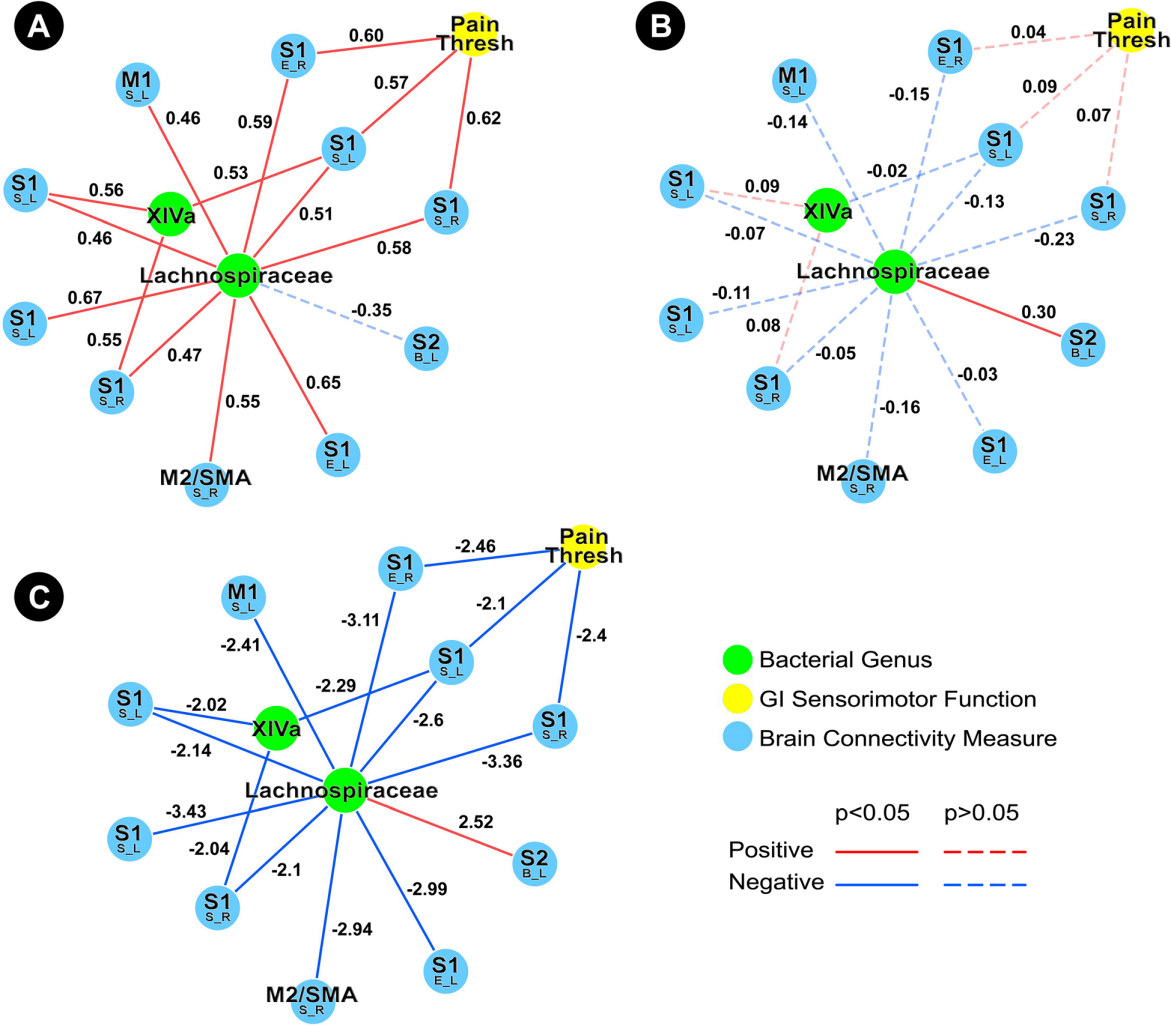
Tripartite *Clostridium XIVa* subnetwork. **a** Demonstrates the *Clostridium XIVa* healthy controls subnetwork. **b** Demonstrates the *Clostridium XIVa* IBS subnetwork. **c** Demonstrates the *Clostridium XIVa* difference network. Functional brain connectivity of regions of interest is presented with the region of interest noted in a larger font, with the connectivity measure and lateralization indicated below in the form X_Y, where *X* indicates a connectivity measure (*B* betweenness centrality, *E* eigenvector centrality, *S* degree strength) and *Y* indicates lateralization (*L* left, *R* right). *Disc Thresh* discomfort threshold during balloon distension, *LC Pain* pain during lactulose challenge test, *OATT* oroanal transit time, *Pain Thresh* pain threshold during balloon distension, *Disc Intensity* visual analogue scale rating of discomfort during 24 mmHg distension, *XIVa Clostridium XIVa*, *Lachnospiraceae Lachnospiraceae incertae sedis*

The HC subnetwork showed indirect associations between *Clostridium XIVa* and several measures of visceral sensitivity, including rectal discomfort threshold (through connectivity of the putamen), rectal discomfort intensity (through connectivity of S1), rectal pain threshold (through connectivity of the putamen, NAcc, and S1), and pain during the nutrient and lactulose challenge test (through connectivity of S1). Indirect associations were also observed for OATT (through connectivity of the putamen and NAcc) (Fig. 4a). In contrast, the IBS subnetwork did not demonstrate any associations between *Clostridium XIVa* and any measures of GI sensorimotor function. However, the IBS subnetwork did show exclusively positive associations between *Clostridium XIVa* and connectivity of subcortical regions (putamen, caudate, and thalamus) (Fig. 4b). The *Clostridium XIVa* difference network underscores that the observed differences are significant (Fig. 4c). *Coprococcus* and *Lachnospiraceae incertae sedis* were also a part of the *Clostridium XIVa* subnetwork.

#### Clostridium XIVb

**Fig. 5:**
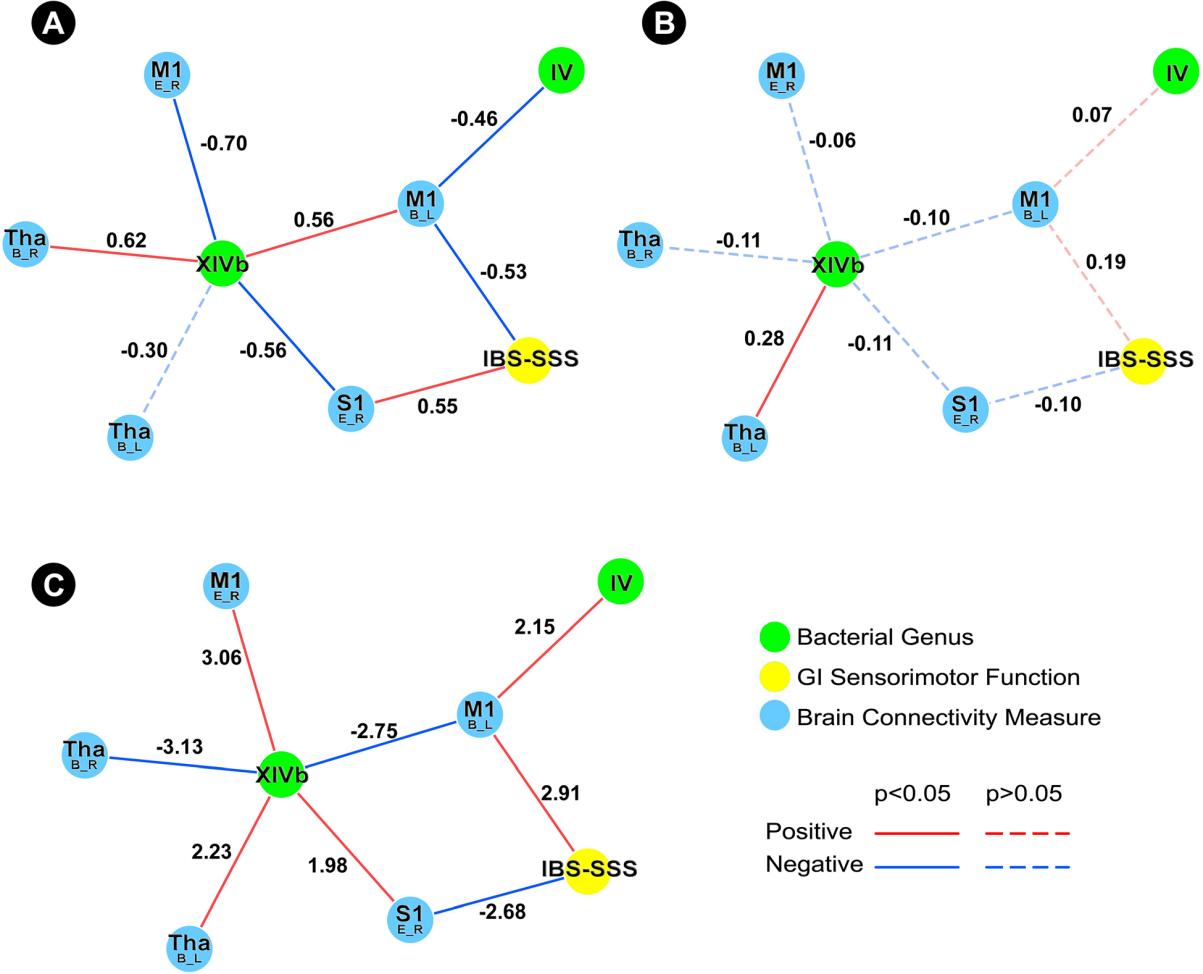
Tripartite *Clostridium XIVb* subnetwork. **a** Demonstrates the *Clostridium XIVb* healthy controls subnetwork. **b** Demonstrates the *Clostridium XIVb* IBS subnetwork. **c** Demonstrates the *Clostridium XIVb* difference network. Functional brain connectivity of regions of interest is presented with the region of interest noted in a larger font, with the connectivity measure and lateralization indicated below in the form X_Y, where *X* indicates a connectivity measure (*B* betweenness centrality, *E* eigenvector centrality, *S* degree strength) and *Y* indicates lateralization (*L* left, *R* right). *IBS*-*SSS* IBS severity scoring system, *IV Clostridium IV*, *XIVb Clostridium XIVb*

The HCs subnetwork showed an indirect association between *Clostridium XIVb* and IBS-SSS through connectivity of S1 and M1. Additionally, *Clostridium IV*, which was also a part of the *Clostridium XIVb* subnetwork, also showed an indirect association with IBS-SSS through connectivity of the same region of M1 (Fig. 5a). In contrast, the IBS subnetwork did not demonstrate any associations between *Clostridium XIVb* and IBS-SSS, but did show a positive association between this genus and connectivity of the thalamus (Fig. 5b). The *Clostridium XIVb* difference network underscores that the observed differences are significant (Fig. 5c).

## Discussion

In this study, we demonstrate differences in microbial subnetworks of IBS patients and HCs that may reflect alterations in the interactions within the brain-gut-microbiome axis of IBS subjects. Our tripartite analysis both supports previous neuroimaging studies in this patient population, and also demonstrates novel findings that may provide the foundation for future, larger, mechanistic studies. As we have previously shown in preclinical studies that members of the order Clostridiales (particularly enriched with families *Ruminococcaceae* and *Lachnospiraceae*) stimulate the biosynthesis and release of 5-HT from intestinal enterochromaffin cells and modulate GI motility, we focused exclusively on genera from this subset of microbes [12]. Although these results were obtained in a relatively small study population, and will need to be confirmed in a much larger study, to our knowledge, this is the first study demonstrating differences between microbial genera subnetworks in IBS patients and HCs with respect to functional connectivity of brain regions in the somatosensory network and GI sensorimotor function.

### Tripartite analysis reveals disruptions in IBS network architecture

Overall, the HC subnetworks demonstrate numerous associations with GI sensorimotor function, which were absent from the IBS group. In HCs, the subnetworks of *Lachnospiraceae incertae sedis*, *Clostridium XIVa*, and *Coprococcus*, in particular, demonstrate robust interactions between their respective microbial genera and GI sensorimotor function through connectivity of regions within the somatosensory network. This is in contrast to the less robust subnetworks in IBS subjects, which are characterized by a loss of these, perhaps protective, associations.

In HCs, the *Lachnospiraceae incertae sedis* subnetwork demonstrates indirect associations between *Lachnospiraceae incertae sedis* and rectal pain threshold through connectivity of S1. In contrast, the IBS subnetwork is significantly less robust, showing no associations, direct or indirect, between this genus and any measure of GI sensorimotor function. However, in contrast to the numerous positive associations between *Lachnospiraceae incertae sedis* and S1 in HCs, the IBS subnetwork showed a positive association between this genus and connectivity of S2. Though both S1 and S2 are involved in processing painful sensorimotor input, S1 has been documented to play an important role in the localization and discrimination of painful and noxious stimuli intensity, whereas S2 performs higher order functions involving sensorimotor integration, attention, learning, and memory related to pain [39–41]. The differences in *Lachnospiraceae incertae sedis* subnetworks between the HCs and IBS reported here are supported by previous studies that show altered activity in S1 and S2, as well as an association of these alterations with visceral sensitivity in fMRI studies of IBS patients [42]. Although no conclusions about mechanisms underlying the observed differences in the *Lachnospiraceae incertae sedis* subnetworks (or subnetworks involving other genera) can be made from the current cross sectional study, a plausible explanation involves a disruption in this genus’ ability to modulate the host serotonergic system. In support of this, one study found that *Lachnospiraceae incertae sedis* was increased in patients with diarrhea-predominant IBS, a subgroup that has been associated with increased plasma 5-HT levels [43, 44].

Similar to the *Lachnospiraceae incertae sedis* subnetworks, the *Clostridium XIVa* and *Coprococcus* subnetworks show numerous indirect associations between these genera and GI sensorimotor function in HCs, but not in IBS. Many of these indirect associations involve connectivity of subcortical brain regions. More specifically, the *Clostridium XIVa* HC subnetwork demonstrates indirect associations between *Clostridium XIVa* with rectal pain threshold (through connectivity of the putamen, NAcc, and S1), rectal discomfort threshold (through connectivity of the putamen), rectal discomfort intensity (through connectivity of S1), pain during the nutrient and lactulose challenge test (through connectivity of S2), and OATT (through connectivity of the putamen and NAcc). The *Coprococcus* HC subnetwork demonstrates direct negative associations between *Coprococcus* and pain and discomfort during the nutrient and lactulose challenge test, in addition to indirect associations between *Coprococcus* and pain during this challenge test through connectivity of the caudate. In contrast, the IBS subnetworks are more sparse, with the *Coprococcus* subnetwork showing no meaningful associations. The *Clostridium XIVa* subnetwork does demonstrate exclusively positive associations between *Clostridium XIVa* and connectivity of subcortical regions (putamen, caudate, and thalamus).

These results align with a previous report from our group, which showed positive associations between Firmicutes-associated Clostridia (such as *Coprococcus* and *Clostridium XIVa*) and increased gray matter volume of the putamen, caudate, and nucleus accumbens in IBS patients [11]. Numerous studies have demonstrated activation of subcortical regions, in particular the putamen, during both acute and chronic pain [45, 46]. Additionally, previous fMRI studies involving experimental rectal stimulation have demonstrated a role for the basal ganglia in IBS-specific alterations in pain processing [4, 47]. One study identified microstructural reorganization in basal ganglia regions in patients with IBS [48]. Deep brain stimulation of the caudate nucleus in a patient with obsessive compulsive disorder and IBS reduced both psychiatric and gastrointestinal symptoms [49]. *Coprococcus* and *Clostridium XIVa* may contribute to visceral hypersensitivity and pain in IBS by influencing these subcortical regions.

In HCs, *Clostridium XIVb* and *Clostridium IV* showed indirect associations with IBS-SSS through connectivity of cortical regions (M1 and S1). In IBS, although *Clostridium XIVb* showed an indirect association with IBS-SSS through connectivity of the thalamus, this association did not emerge as significant with respect to the difference network. Interestingly, IBS-SSS did not show more numerous associations with microbial genera in the IBS subnetworks. A previous study, which identified an intestinal microbiota signature associated with severity of IBS, suggested an important role for operational taxonomic units/“species” in the genera *Roseburia*, *Lachnospiraceae incertae sedis*, and *Clostridium XIVa* in IBS symptom severity [10]. The difference in our results from this previous study could be attributed to differences in methodology and study design, as the aforementioned study used machine learning approaches and operational taxonomic units, rather than genera.

### Limitations

This study focused on microorganisms and brain regions that have previously been implicated in IBS pathophysiology. The specific microorganisms have been shown in preclinical studies to modulate host serotonin biosynthesis and release in the GI tract and to be associated with elevated relative abundances in subsets of patients with IBS [11, 12]. Future studies in larger populations may benefit from expanding the genera we have investigated, or alternatively, exploring the effects of individual species or operational taxonomic units. Due to the limited sample size, we did not investigate the influence of psychiatric variables (anxiety, depression) or history of early adversity that may confound the findings presented in this study. Due to the small sample size and the exploratory nature of this study, correction for multiple comparisons was not performed. Alternative analytical approaches to data analysis, perhaps involving methods specific for operational taxonomical unit (OTU)-OTU correlation networks rather than for abundance data correlation networks, should be explored in future work. Although future, larger studies of this type may reveal differences in certain components of the subnetworks we have presented here, it is likely that the overall patterns and differences in subnetworks between IBS and HCs will remain. As the microorganisms investigated in this study have been implicated in serotonergic modulation, future studies should incorporate measurements of 5-HT and other tryptophan metabolites in their investigation of BGM interactions in IBS. As this is an association study, no causative relationships can be implied, and the directionality of the interactions between relative microbial abundance and brain connectivity cannot be parsed out. However, previous work has suggested a bidirectional model for brain-gut-microbiome communication, with evidence for both top-down and bottom-up communication as playing an important role in shaping the IBS and HCs subnetworks we have demonstrated. [50]

## Conclusions

In this study, we build on our previous work on brain gut microbiome interactions that has suggested a role for microorganisms in the order Clostridiales in modulating host 5-HT biosynthesis and release, and influencing brain regions in patients with IBS. To our knowledge, this is the first study investigating differences between IBS and HCs with respect to the interaction network between microorganisms, functional connectivity of brain regions in the somatosensory network, and GI sensorimotor function. Our results suggest that disruptions in the brain-gut-microbiome axis in IBS patients involving mainly subcortical but also cortical brain regions may contribute to visceral hypersensitivity and altered perception of pain in patients with IBS.

## Additional files

Additional file 1: **Table S1.** Healthy Controls Network Associations. This table shows all of the associations of the healthy controls network. Functional connectivity of regions of interest are presented in the format: X_Y_Z, where X indicates a connectivity measure (B, Betweenness centrality; E, Eigenvector centrality; S, Degree strength), Y indicates lateralization (L, Left; R, Right), and Z indicates a region of interest (see Table 1). Abbreviations: First Rectal Sensation, first sensation threshold during balloon distension; Rectal Discomfort Threshold, discomfort threshold during balloon distension; IBS-SS, Irritable Bowel Syndrome - Scoring System scores; Lactulose - Discomfort, discomfort during lactulose challenge test; Lactulose - Pain, pain during lactulose challenge test; OATT, oroanal transit time; Rectal Pain Threshold, pain threshold during balloon distension; Rectal Discomfort Intensity, visual analogue scale rating of discomfort during 24 mmHg distension; Rectal Pain Intensity, visual analogue scale rating of pain during 24 mmHg distension. (DOCX 35 kb)

Additional file 2: **Table S2.** IBS Network Associations. This table shows all of the associations of the IBS network. Functional connectivity of regions of interest are presented in the format: X_Y_Z, where X indicates a connectivity measure (B, Betweenness centrality; E, Eigenvector centrality; S, Degree strength), Y indicates lateralization (L, Left; R, Right), and Z indicates a region of interest (see Table 1). Abbreviations: First Rectal Sensation, first sensation threshold during balloon distension; Rectal Discomfort Threshold, discomfort threshold during balloon distension; IBS-SS, Irritable Bowel Syndrome - Scoring System scores; Lactulose - Discomfort, discomfort during lactulose challenge test; Lactulose - Pain, pain during nutrient and lactulose challenge test; OATT, oroanal transit time; Rectal Pain Threshold, pain threshold during balloon distension; Rectal Discomfort Intensity, visual analogue scale rating of discomfort during 24 mmHg distension; Rectal Pain Intensity, visual analogue scale rating of pain during 24 mmHg distension (DOCX 150 kb)

Additional file 3: **Table S3.** Difference Network Z Tests. This table shows all of the Z tests that define the difference network. Functional connectivity of regions of interest are presented in the format: X_Y_Z, where X indicates a connectivity measure (B, Betweenness centrality; E, Eigenvector centrality; S, Degree strength), Y indicates lateralization (L, Left; R, Right), and Z indicates a region of interest (see Table 1). Abbreviations: First Rectal Sensation, first sensation threshold during balloon distension; Rectal Discomfort Threshold, discomfort threshold during balloon distension; IBS-SS, Irritable Bowel Syndrome - Scoring System scores; Lactulose - Discomfort, discomfort during nutrient and lactulose challenge test; Lactulose - Pain, pain during lactulose challenge test; OATT, oroanal transit time; Rectal Pain Threshold, pain threshold during balloon distension; Rectal Discomfort Intensity, visual analogue scale rating of discomfort during 24 mmHg distension; Rectal Pain Intensity, visual analogue scale rating of pain during 24 mmHg distension. DOCX 156 kb)

Additional file 4: Supplemental Materials. Supplemental materials related to statistical power calculations. (DOCX 71 kb)

## Abbreviations

5-HT: Serotonin
CNS: Central nervous system
FODMAP: Fermentable oligosaccharides, disaccharides, monosaccharides, and polyols
GI: Gastrointestinal
HC: Healthy control
IBS: Irritable bowel syndrome
IBS-SSS: IBS severity scoring system
NAcc: Nucleus accumbens
