# Supplementary material for: Evidence for an association of gut microbial Clostridia with brain functional connectivity and gastrointestinal sensorimotor function in patients with irritable bowel syndrome, based on tripartite network analysis
Source: Microbiome. 2019 Mar 21;7:45. doi: 10.1186/s40168-019-0656-z (PMC6429755; doi:10.1186/s40168-019-0656-z)
Supplement: Supplementary file 1 — Table S1. Healthy Controls Network Associations. This table shows all of the associations of the healthy controls network. Functional connectivity of regions of interest are presented in the format: X_Y_Z, where X indicates a connectivity measure (B, Betweenness centrality; E, Eigenvector centrality; S, Degree strength), Y indicates lateralization (L, Left; R, Right), and Z indicates a region of interest (see Table 1). Abbreviations: First Rectal Sensation, first sensation threshold during balloon distension; Rectal Discomfort Threshold, discomfort threshold during balloon distension; IBS-SS, Irritable Bowel Syndrome - Scoring System scores; Lactulose - Discomfort, discomfort during lactulose challenge test; Lactulose - Pain, pain during lactulose challenge test; OATT, oroanal transit time; Rectal Pain Threshold, pain threshold during balloon distension; Rectal Discomfort Intensity, visual analogue scale rating of discomfort during 24 mmHg distension; Rectal Pain Intensity, visual analogue scale rating of pain during 24 mmHg distension. (DOCX 35 kb) [file 40168_2019_656_MOESM1_ESM.docx]

**Table S1. Healthy Controls Network Associations**

| **Microbial Genus** | **Functional Connectivity** | ***r*** | ***p*** | ***df*** |
| --- | --- | --- | --- | --- |
| *Blautia* | B_L_SupFS | 0.5821 | 0.0089 | 20 |
| *Blautia* | B_R_SupFS | 0.5070 | 0.0267 | 20 |
| *Blautia* | B_L_InfPrCS | -0.5055 | 0.0273 | 20 |
| *Blautia* | B_L_SupPrCs | -0.4900 | 0.0332 | 20 |
| *Blautia* | B_R_InfPrCS | -0.4682 | 0.0432 | 20 |
| *Blautia* | B_R_LoInG_CInS | -0.3956 | 0.0937 | 20 |
| *Blautia* | B_R_InfCirInS | -0.3950 | 0.0942 | 20 |
| *Blautia* | B_R_SupCirInS | -0.3790 | 0.1096 | 20 |
| *Blautia* | E_R_LoInG_CInS | -0.2404 | 0.3214 | 20 |
| *Blautia* | E_R_InfCirInS | -0.2329 | 0.3373 | 20 |
| *Blautia* | B_L_InfPrCS | -0.0260 | 0.9157 | 20 |
| *Clostridium IV* | B_R_InfCirInS | 0.5598 | 0.0127 | 20 |
| *Clostridium IV* | B_L_PRCG | -0.5187 | 0.0229 | 20 |
| *Clostridium IV* | B_L_SupPrCs | -0.4641 | 0.0453 | 20 |
| *Clostridium IV* | B_L_InfCirInS | 0.4625 | 0.0461 | 20 |
| *Clostridium IV* | B_R_PosCG | -0.3651 | 0.1243 | 20 |
| *Clostridium IV* | B_L_SupFG | -0.3405 | 0.1537 | 20 |
| *Clostridium IV* | B_R_Tha | 0.0222 | 0.9281 | 20 |
| *Clostridium XlVa* | B_L_PosCS | -0.6585 | 0.0022 | 20 |
| *Clostridium XlVa* | B_L_SbCG_S | 0.5953 | 0.0072 | 20 |
| *Clostridium XlVa* | B_R_PosCG | 0.5906 | 0.0078 | 20 |
| *Clostridium XlVa* | B_L_CS | 0.5599 | 0.0127 | 20 |
| *Clostridium XlVa* | B_R_CS | 0.5528 | 0.0141 | 20 |
| *Clostridium XlVa* | B_R_SbCG_S | 0.5464 | 0.0155 | 20 |
| *Clostridium XlVa* | B_L_Pu | -0.5422 | 0.0165 | 20 |
| *Clostridium XlVa* | B_L_PosCG | 0.5337 | 0.0186 | 20 |
| *Clostridium XlVa* | B_L_Nacc | 0.5308 | 0.0194 | 20 |
| *Clostridium XlVa* | B_R_PRCG | 0.5226 | 0.0217 | 20 |
| *Clostridium XlVa* | B_L_PosLS | 0.5086 | 0.0262 | 20 |
| *Clostridium XlVa* | B_L_InfCirInS | 0.4741 | 0.0403 | 20 |
| *Clostridium XlVa* | B_L_PRCG | 0.4620 | 0.0464 | 20 |
| *Clostridium XlVa* | E_L_Pu | -0.3615 | 0.1284 | 20 |
| *Clostridium XlVa* | E_R_Pu | -0.3482 | 0.1440 | 20 |
| *Clostridium XlVa* | E_L_CaN | -0.3283 | 0.1699 | 20 |
| *Clostridium XlVa* | B_L_Tha | -0.2588 | 0.2847 | 20 |
| *Clostridium XlVb* | E_R_PRCG | -0.7048 | 0.0008 | 20 |
| *Clostridium XlVb* | B_R_Tha | 0.6239 | 0.0043 | 20 |
| *Clostridium XlVb* | B_L_SupPrCs | 0.5638 | 0.0119 | 20 |
| *Clostridium XlVb* | E_R_CS | -0.5625 | 0.0122 | 20 |
| *Clostridium XlVb* | E_L_SbCG_S | -0.5463 | 0.0155 | 20 |
| *Clostridium XlVb* | E_L_SupFS | 0.4939 | 0.0316 | 20 |
| *Clostridium XlVb* | B_R_LoInG_CInS | -0.4883 | 0.0339 | 20 |
| *Clostridium XlVb* | B_R_PosLS | 0.4800 | 0.0375 | 20 |
| *Clostridium XlVb* | B_R_InfCirInS | -0.4599 | 0.0476 | 20 |
| *Clostridium XlVb* | B_L_Tha | -0.2976 | 0.2160 | 20 |
| *Clostridium XlVb* | E_L_Nacc | 0.2535 | 0.2950 | 20 |
| *Clostridium XlVb* | E_R_CaN | 0.2217 | 0.3616 | 20 |
| *Clostridium XlVb* | E_L_Tha | 0.1521 | 0.5343 | 20 |
| *Clostridium XlVb* | B_L_Tha | 0.0823 | 0.7378 | 20 |
| *Clostridium XlVb* | B_R_PRCG | 0.0531 | 0.8291 | 20 |
| *Clostridium XlVb* | E_R_Pu | -0.0342 | 0.8895 | 20 |
| *Clostridium XlVb* | B_R_Pu | -0.0225 | 0.9270 | 20 |
| *Coprococcus* | B_L_CaN | -0.6067 | 0.0059 | 20 |
| *Coprococcus* | E_R_CaN | -0.5810 | 0.0091 | 20 |
| *Coprococcus* | E_L_CaN | -0.5685 | 0.0111 | 20 |
| *Coprococcus* | B_L_SbCG_S | 0.5579 | 0.0131 | 20 |
| *Coprococcus* | B_R_PosCG | -0.5085 | 0.0262 | 20 |
| *Coprococcus* | B_R_SbCG_S | 0.5072 | 0.0267 | 20 |
| *Coprococcus* | B_L_PosLS | 0.5042 | 0.0277 | 20 |
| *Coprococcus* | B_R_CaN | -0.5026 | 0.0283 | 20 |
| *Coprococcus* | B_R_InfCirInS | -0.4573 | 0.0490 | 20 |
| *Coprococcus* | E_L_InfPrCS | -0.3218 | 0.1791 | 20 |
| *Coprococcus* | B_L_SupFS | 0.2754 | 0.2538 | 20 |
| *Coprococcus* | E_L_SupFS | -0.0596 | 0.8086 | 20 |
| *Faecalibacterium* | B_R_PosLS | 0.4938 | 0.0317 | 20 |
| *Faecalibacterium* | E_R_PRCG | -0.4854 | 0.0352 | 20 |
| *Lachnospiraceae incertae sedis* | B_L_PosCS | 0.6684 | 0.0018 | 20 |
| *Lachnospiraceae incertae sedis* | E_L_PosCS | 0.6465 | 0.0028 | 20 |
| *Lachnospiraceae incertae sedis* | E_R_PosCS | 0.5906 | 0.0078 | 20 |
| *Lachnospiraceae incertae sedis* | B_R_PosCS | 0.5835 | 0.0087 | 20 |
| *Lachnospiraceae incertae sedis* | B_R_SupFS | 0.5528 | 0.0141 | 20 |
| *Lachnospiraceae incertae sedis* | B_L_PosCG | 0.5128 | 0.0247 | 20 |
| *Lachnospiraceae incertae sedis* | B_R_CS | 0.4699 | 0.0424 | 20 |
| *Lachnospiraceae incertae sedis* | B_R_SupFG | 0.4623 | 0.0463 | 20 |
| *Lachnospiraceae incertae sedis* | B_L_PRCG | 0.4612 | 0.0469 | 20 |
| *Lachnospiraceae incertae sedis* | B_L_CS | 0.4609 | 0.0470 | 20 |
| *Lachnospiraceae incertae sedis* | B_R_SupFG | 0.4245 | 0.0701 | 20 |
| *Lachnospiraceae incertae sedis* | B_L_SbCG_S | -0.3532 | 0.1380 | 20 |
| *Lachnospiraceae incertae sedis* | B_L_SupFG | -0.1615 | 0.5088 | 20 |
| *Lachnospiraceae incertae sedis* | B_L_InfCirInS | 0.1025 | 0.6762 | 20 |
| *Lachnospiraceae incertae sedis* | B_R_PosLS | 0.0840 | 0.7325 | 20 |
| *Lachnospiraceae incertae sedis* | B_R_Nacc | -0.0531 | 0.8292 | 20 |
| *Lachnospiraceae incertae sedis* | B_R_SbCG_S | -0.0511 | 0.8354 | 20 |
| *Lachnospiraceae incertae sedis* | B_L_InfPrCS | -0.0238 | 0.9230 | 20 |
| *Oscillibacter* | E_L_InfCirInS | 0.4640 | 0.0454 | 20 |
| *Oscillibacter* | B_L_CaN | -0.2769 | 0.2511 | 20 |
| *Oscillibacter* | B_R_SupCirInS | -0.1393 | 0.5696 | 20 |
| *Oscillibacter* | B_R_InfPrCS | 0.0841 | 0.7321 | 20 |
| *Oscillibacter* | B_R_PosLS | 0.0428 | 0.8620 | 20 |
| *Oscillibacter* | B_L_LoInG_CInS | -0.0401 | 0.8706 | 20 |
| *Roseburia* | B_R_SupFS | 0.4830 | 0.0362 | 20 |
| *Roseburia* | B_R_Pu | -0.4654 | 0.0446 | 20 |
| *Roseburia* | B_L_CaN | 0.4651 | 0.0448 | 20 |
| *Roseburia* | B_R_SupCirInS | 0.3107 | 0.1954 | 20 |
| *Roseburia* | E_R_LoInG_CInS | -0.2080 | 0.3928 | 20 |
| *Roseburia* | B_R_LoInG_CInS | -0.1908 | 0.4339 | 20 |
| *Roseburia* | B_L_PosLS | 0.1828 | 0.4538 | 20 |
| *Roseburia* | E_L_Pal | -0.1683 | 0.4909 | 20 |
| *Roseburia* | B_R_LoInG_CInS | -0.0239 | 0.9227 | 20 |
|  |  |  |  |  |
| **Microbial Genus** | **Clinical Measure** | ***r*** | ***p*** | ***df*** |
| *Clostridium IV* | Rectal Discomfort Intensity | 0.2510 | 0.2999 | 20 |
| *Clostridium XlVa* | Lactulose - Pain | 0.0048 | 0.9846 | 20 |
| *Clostridium XlVa* | Rectal Pain Threshold | 0.4930 | 0.0320 | 20 |
| *Clostridium XlVb* | Lactulose - Pain | -0.0219 | 0.9290 | 20 |
| *Coprococcus* | Lactulose - Discomfort | -0.5564 | 0.0134 | 20 |
| *Coprococcus* | Lactulose - Pain | -0.6151 | 0.0051 | 20 |
| *Oscillibacter* | First Rectal Sensation | 0.0304 | 0.9016 | 20 |
| *Roseburia* | Lactulose - Discomfort | 0.1745 | 0.4749 | 20 |
|  |  |  |  |  |
| **Clinical Measure** | **Functional Connectivity** | ***r*** | ***p*** | ***df*** |
| First Rectal Sensation | B_L_SupFS | 0.5371 | 0.0177 | 20 |
| First Rectal Sensation | B_R_SupFS | 0.4900 | 0.0332 | 20 |
| First Rectal Sensation | B_L_InfCirInS | -0.2021 | 0.4066 | 20 |
| First Rectal Sensation | B_L_PRCG | -0.1934 | 0.4275 | 20 |
| First Rectal Sensation | B_R_InfCirInS | -0.1775 | 0.4672 | 20 |
| First Rectal Sensation | E_L_InfPrCS | 0.1581 | 0.5181 | 20 |
| First Rectal Sensation | B_R_LoInG_CInS | -0.1342 | 0.5838 | 20 |
| First Rectal Sensation | B_L_Pal | 0.0937 | 0.7027 | 20 |
| IBS-SSS | E_R_CS | 0.5500 | 0.0147 | 20 |
| IBS-SSS | B_L_SupPrCs | -0.5291 | 0.0198 | 20 |
| IBS-SSS | E_L_PosCG | 0.5156 | 0.0238 | 20 |
| IBS-SSS | E_L_SupFS | -0.4807 | 0.0372 | 20 |
| IBS-SSS | E_L_CS | 0.4655 | 0.0446 | 20 |
| IBS-SSS | B_L_SupCirInS | 0.4155 | 0.0769 | 20 |
| IBS-SSS | E_R_Tha | -0.2800 | 0.2456 | 20 |
| IBS-SSS | B_R_Tha | -0.1768 | 0.4689 | 20 |
| IBS-SSS | B_L_Tha | -0.1398 | 0.5681 | 20 |
| IBS-SSS | B_L_PosCG | 0.0486 | 0.8434 | 20 |
| Lactulose - Discomfort | B_R_SbCG_S | -0.5870 | 0.0082 | 20 |
| Lactulose - Discomfort | E_R_SbCG_S | -0.5759 | 0.0099 | 20 |
| Lactulose - Discomfort | B_L_PosCG | 0.5437 | 0.0161 | 20 |
| Lactulose - Discomfort | B_L_SbCG_S | -0.5063 | 0.0270 | 20 |
| Lactulose - Discomfort | B_L_CS | -0.4806 | 0.0373 | 20 |
| Lactulose - Discomfort | B_R_PosLS | -0.4785 | 0.0382 | 20 |
| Lactulose - Discomfort | E_L_SupFS | 0.4751 | 0.0398 | 20 |
| Lactulose - Discomfort | B_R_LoInG_CInS | -0.4614 | 0.0467 | 20 |
| Lactulose - Discomfort | B_R_CS | -0.4295 | 0.0665 | 20 |
| Lactulose - Discomfort | B_L_PRCG | -0.3984 | 0.0911 | 20 |
| Lactulose - Discomfort | B_L_LoInG_CInS | -0.3845 | 0.1040 | 20 |
| Lactulose - Discomfort | B_L_PosCG | -0.3831 | 0.1054 | 20 |
| Lactulose - Discomfort | B_L_PosLS | -0.3804 | 0.1081 | 20 |
| Lactulose - Discomfort | B_R_PosCG | -0.3764 | 0.1122 | 20 |
| Lactulose - Discomfort | B_R_PRCG | -0.3565 | 0.1340 | 20 |
| Lactulose - Discomfort | B_L_PosLS | -0.3396 | 0.1549 | 20 |
| Lactulose - Discomfort | E_R_Tha | 0.2163 | 0.3737 | 20 |
| Lactulose - Discomfort | B_L_PosCS | 0.1844 | 0.4499 | 20 |
| Lactulose - Discomfort | E_L_Tha | 0.1772 | 0.4681 | 20 |
| Lactulose - Discomfort | B_L_SupCirInS | -0.1702 | 0.4860 | 20 |
| Lactulose - Discomfort | E_L_PRCG | -0.0521 | 0.8323 | 20 |
| Lactulose - Discomfort | B_L_Tha | 0.0408 | 0.8682 | 20 |
| Lactulose - Discomfort | B_L_InfCirInS | -0.0196 | 0.9365 | 20 |
| Lactulose - Pain | E_L_CaN | 0.6129 | 0.0053 | 20 |
| Lactulose - Pain | B_L_CaN | 0.5422 | 0.0165 | 20 |
| Lactulose - Pain | B_R_SbCG_S | -0.5329 | 0.0188 | 20 |
| Lactulose - Pain | E_R_CaN | 0.5310 | 0.0193 | 20 |
| Lactulose - Pain | E_R_Nacc | 0.5301 | 0.0196 | 20 |
| Lactulose - Pain | B_L_SbCG_S | -0.5095 | 0.0259 | 20 |
| Lactulose - Pain | E_L_Nacc | 0.5086 | 0.0262 | 20 |
| Lactulose - Pain | B_R_PosLS | -0.4830 | 0.0362 | 20 |
| Lactulose - Pain | B_L_PosLS | -0.4688 | 0.0429 | 20 |
| Lactulose - Pain | B_R_SupFS | 0.2364 | 0.3300 | 20 |
| Lactulose - Pain | E_L_PRCG | 0.2350 | 0.3328 | 20 |
| Lactulose - Pain | B_L_Tha | 0.1508 | 0.5378 | 20 |
| OATT | B_L_Pu | 0.5825 | 0.0089 | 20 |
| OATT | B_L_SupCirInS | -0.5627 | 0.0121 | 20 |
| OATT | B_L_Nacc | -0.5406 | 0.0169 | 20 |
| OATT | B_R_Pu | -0.5350 | 0.0183 | 20 |
| OATT | B_L_PosLS | -0.4819 | 0.0367 | 20 |
| OATT | B_R_SbCG_S | -0.4704 | 0.0421 | 20 |
| OATT | B_R_CS | 0.2301 | 0.3434 | 20 |
| OATT | B_L_SupPrCs | -0.0306 | 0.9011 | 20 |
| Rectal Discomfort Intensity | B_R_CaN | 0.6316 | 0.0037 | 20 |
| Rectal Discomfort Intensity | E_L_InfPrCS | 0.5584 | 0.0130 | 20 |
| Rectal Discomfort Intensity | B_L_PosCS | 0.5248 | 0.0211 | 20 |
| Rectal Discomfort Intensity | E_R_SupCirInS | 0.2241 | 0.3564 | 20 |
| Rectal Discomfort Intensity | B_R_SupCirInS | 0.1898 | 0.4363 | 20 |
| Rectal Discomfort Intensity | E_L_SupCirInS | 0.1247 | 0.6110 | 20 |
| Rectal Discomfort Intensity | B_R_Tha | -0.0381 | 0.8768 | 20 |
| Rectal Discomfort Threshold | B_L_SupCirInS | 0.6121 | 0.0053 | 20 |
| Rectal Discomfort Threshold | B_R_CaN | -0.5205 | 0.0223 | 20 |
| Rectal Discomfort Threshold | B_L_Pu | -0.4854 | 0.0351 | 20 |
| Rectal Pain Intensity | B_R_CaN | 0.5954 | 0.0072 | 20 |
| Rectal Pain Intensity | B_L_Nacc | -0.4870 | 0.0345 | 20 |
| Rectal Pain Intensity | E_L_SupFG | 0.4777 | 0.0386 | 20 |
| Rectal Pain Intensity | B_L_PosCS | 0.4579 | 0.0487 | 20 |
| Rectal Pain Intensity | B_R_PosLS | -0.2849 | 0.2372 | 20 |
| Rectal Pain Intensity | B_L_SupCirInS | -0.2608 | 0.2808 | 20 |
| Rectal Pain Intensity | B_R_SbCG_S | 0.2567 | 0.2888 | 20 |
| Rectal Pain Intensity | B_L_Tha | 0.2503 | 0.3014 | 20 |
| Rectal Pain Intensity | B_R_PRCG | -0.1548 | 0.5270 | 20 |
| Rectal Pain Intensity | E_R_PosLS | -0.1313 | 0.5921 | 20 |
| Rectal Pain Intensity | B_R_Tha | 0.0691 | 0.7787 | 20 |
| Rectal Pain Intensity | B_R_PRCG | -0.0539 | 0.8267 | 20 |
| Rectal Pain Intensity | B_R_InfPrCS | -0.0294 | 0.9048 | 20 |
| Rectal Pain Intensity | B_L_SupFS | 0.0223 | 0.9279 | 20 |
| Rectal Pain Threshold | E_L_CS | 0.6737 | 0.0016 | 20 |
| Rectal Pain Threshold | B_R_LoInG_CInS | -0.6685 | 0.0018 | 20 |
| Rectal Pain Threshold | E_L_Tha | -0.6471 | 0.0027 | 20 |
| Rectal Pain Threshold | B_L_Pu | -0.6379 | 0.0033 | 20 |
| Rectal Pain Threshold | B_R_PosCG | 0.6274 | 0.0040 | 20 |
| Rectal Pain Threshold | B_R_PosCS | 0.6159 | 0.0050 | 20 |
| Rectal Pain Threshold | E_R_PosCS | 0.6042 | 0.0061 | 20 |
| Rectal Pain Threshold | B_R_CS | 0.5967 | 0.0070 | 20 |
| Rectal Pain Threshold | B_L_Nacc | 0.5792 | 0.0094 | 20 |
| Rectal Pain Threshold | B_L_PosCG | 0.5728 | 0.0104 | 20 |
| Rectal Pain Threshold | E_R_PosCG | 0.5630 | 0.0121 | 20 |
| Rectal Pain Threshold | B_L_CS | 0.5610 | 0.0125 | 20 |
| Rectal Pain Threshold | E_R_Tha | -0.5344 | 0.0184 | 20 |
| Rectal Pain Threshold | B_L_PosCS | -0.5246 | 0.0211 | 20 |
| Rectal Pain Threshold | B_L_SbCG_S | 0.5167 | 0.0235 | 20 |
| Rectal Pain Threshold | E_R_CS | 0.4901 | 0.0331 | 20 |
| Rectal Pain Threshold | E_L_Pu | -0.4899 | 0.0332 | 20 |
| Rectal Pain Threshold | E_L_PosCG | 0.4883 | 0.0339 | 20 |
| Rectal Pain Threshold | B_L_PRCG | 0.4837 | 0.0359 | 20 |
| Rectal Pain Threshold | B_R_SbCG_S | 0.4818 | 0.0367 | 20 |
| Rectal Pain Threshold | E_R_CaN | -0.4735 | 0.0406 | 20 |
| Rectal Pain Threshold | B_R_PosLS | 0.4734 | 0.0406 | 20 |
| Rectal Pain Threshold | B_L_SupFS | 0.0428 | 0.8618 | 20 |
