# Supplementary material for: Evidence for an association of gut microbial Clostridia with brain functional connectivity and gastrointestinal sensorimotor function in patients with irritable bowel syndrome, based on tripartite network analysis
Source: Microbiome. 2019 Mar 21;7:45. doi: 10.1186/s40168-019-0656-z (PMC6429755; doi:10.1186/s40168-019-0656-z)
Supplement: Supplementary file 2 — Table S2. IBS Network Associations. This table shows all of the associations of the IBS network. Functional connectivity of regions of interest are presented in the format: X_Y_Z, where X indicates a connectivity measure (B, Betweenness centrality; E, Eigenvector centrality; S, Degree strength), Y indicates lateralization (L, Left; R, Right), and Z indicates a region of interest (see Table 1). Abbreviations: First Rectal Sensation, first sensation threshold during balloon distension; Rectal Discomfort Threshold, discomfort threshold during balloon distension; IBS-SS, Irritable Bowel Syndrome - Scoring System scores; Lactulose - Discomfort, discomfort during lactulose challenge test; Lactulose - Pain, pain during nutrient and lactulose challenge test; OATT, oroanal transit time; Rectal Pain Threshold, pain threshold during balloon distension; Rectal Discomfort Intensity, visual analogue scale rating of discomfort during 24 mmHg distension; Rectal Pain Intensity, visual analogue scale rating of pain during 24 mmHg distension (DOCX 150 kb) [file 40168_2019_656_MOESM2_ESM.docx]

**Table S2. IBS Network Associations**

| **Microbial Genus** | **Functional Connectivity** | ***r*** | ***p*** | ***df*** |
| --- | --- | --- | --- | --- |
| *Blautia* | B_R_InfCirInS | -0.2928 | 0.0199 | 64 |
| *Blautia* | B_L_InfPrCS | -0.2799 | 0.0263 | 64 |
| *Blautia* | B_R_SupCirInS | -0.2560 | 0.0428 | 64 |
| *Blautia* | E_R_LoInG_CInS | -0.2557 | 0.0431 | 64 |
| *Blautia* | B_R_LoInG_CInS | -0.2544 | 0.0443 | 64 |
| *Blautia* | E_R_InfCirInS | -0.2538 | 0.0447 | 64 |
| *Blautia* | B_R_InfPrCS | -0.2306 | 0.0691 | 64 |
| *Blautia* | B_L_InfPrCS | -0.1774 | 0.1641 | 64 |
| *Blautia* | B_R_SupFS | 0.1292 | 0.3129 | 64 |
| *Blautia* | B_L_SupPrCs | -0.1157 | 0.3664 | 64 |
| *Blautia* | B_L_SupFS | -0.0857 | 0.5044 | 64 |
| *Clostridium IV* | B_L_SupFG | -0.4366 | 0.0003 | 64 |
| *Clostridium IV* | B_R_Tha | 0.3148 | 0.0120 | 64 |
| *Clostridium IV* | B_R_PosCG | 0.2684 | 0.0334 | 64 |
| *Clostridium IV* | B_L_PRCG | -0.1254 | 0.3275 | 64 |
| *Clostridium IV* | B_L_SupPrCs | 0.0728 | 0.5706 | 64 |
| *Clostridium IV* | B_R_InfCirInS | -0.0609 | 0.6352 | 64 |
| *Clostridium IV* | B_L_InfCirInS | -0.0511 | 0.6907 | 64 |
| *Clostridium XlVa* | E_L_Pu | 0.3022 | 0.0161 | 64 |
| *Clostridium XlVa* | E_R_Pu | 0.2869 | 0.0226 | 64 |
| *Clostridium XlVa* | E_L_CaN | 0.2737 | 0.0300 | 64 |
| *Clostridium XlVa* | B_L_Tha | 0.2680 | 0.0337 | 64 |
| *Clostridium XlVa* | B_L_Pu | 0.1573 | 0.2184 | 64 |
| *Clostridium XlVa* | B_L_PosLS | 0.1121 | 0.3817 | 64 |
| *Clostridium XlVa* | B_L_CS | 0.0917 | 0.4748 | 64 |
| *Clostridium XlVa* | B_R_CS | 0.0758 | 0.5548 | 64 |
| *Clostridium XlVa* | B_R_PRCG | 0.0701 | 0.5851 | 64 |
| *Clostridium XlVa* | B_L_PosCS | -0.0600 | 0.6406 | 64 |
| *Clostridium XlVa* | B_L_Nacc | 0.0577 | 0.6531 | 64 |
| *Clostridium XlVa* | B_L_InfCirInS | 0.0564 | 0.6607 | 64 |
| *Clostridium XlVa* | B_R_SbCG_S | 0.0289 | 0.8219 | 64 |
| *Clostridium XlVa* | B_L_SbCG_S | 0.0217 | 0.8658 | 64 |
| *Clostridium XlVa* | B_L_PRCG | 0.0197 | 0.8784 | 64 |
| *Clostridium XlVa* | B_L_PosCG | -0.0174 | 0.8924 | 64 |
| *Clostridium XlVa* | B_R_PosCG | 0.0075 | 0.9534 | 64 |
| *Clostridium XlVb* | B_R_PRCG | 0.3192 | 0.0108 | 64 |
| *Clostridium XlVb* | E_L_Nacc | 0.3111 | 0.0131 | 64 |
| *Clostridium XlVb* | E_L_Tha | 0.2934 | 0.0196 | 64 |
| *Clostridium XlVb* | E_R_Pu | 0.2924 | 0.0200 | 64 |
| *Clostridium XlVb* | B_L_Tha | 0.2857 | 0.0232 | 64 |
| *Clostridium XlVb* | B_L_Tha | 0.2829 | 0.0247 | 64 |
| *Clostridium XlVb* | E_R_CaN | 0.2680 | 0.0337 | 64 |
| *Clostridium XlVb* | B_R_Pu | 0.2673 | 0.0342 | 64 |
| *Clostridium XlVb* | E_L_SbCG_S | -0.2228 | 0.0792 | 64 |
| *Clostridium XlVb* | E_L_SupFS | 0.2204 | 0.0826 | 64 |
| *Clostridium XlVb* | B_R_InfCirInS | -0.1636 | 0.2001 | 64 |
| *Clostridium XlVb* | B_R_LoInG_CInS | -0.1385 | 0.2791 | 64 |
| *Clostridium XlVb* | E_R_CS | -0.1057 | 0.4097 | 64 |
| *Clostridium XlVb* | B_R_Tha | -0.1051 | 0.4124 | 64 |
| *Clostridium XlVb* | B_L_SupPrCs | -0.0967 | 0.4508 | 64 |
| *Clostridium XlVb* | E_R_PRCG | -0.0587 | 0.6478 | 64 |
| *Clostridium XlVb* | B_R_PosLS | 0.0184 | 0.8859 | 64 |
| *Coprococcus* | E_L_InfPrCS | -0.3424 | 0.0060 | 64 |
| *Coprococcus* | B_L_SupFS | 0.2681 | 0.0336 | 64 |
| *Coprococcus* | E_L_SupFS | 0.2499 | 0.0482 | 64 |
| *Coprococcus* | E_R_CaN | -0.1502 | 0.2400 | 64 |
| *Coprococcus* | B_R_CaN | -0.0951 | 0.4584 | 64 |
| *Coprococcus* | B_L_PosLS | -0.0884 | 0.4909 | 64 |
| *Coprococcus* | B_L_SbCG_S | 0.0725 | 0.5721 | 64 |
| *Coprococcus* | B_R_SbCG_S | 0.0684 | 0.5940 | 64 |
| *Coprococcus* | E_L_CaN | -0.0519 | 0.6861 | 64 |
| *Coprococcus* | B_L_CaN | -0.0417 | 0.7456 | 64 |
| *Coprococcus* | B_R_InfCirInS | -0.0376 | 0.7696 | 64 |
| *Coprococcus* | B_R_PosCG | 0.0127 | 0.9215 | 64 |
| *Faecalibacterium* | B_R_PosLS | -0.1731 | 0.1748 | 64 |
| *Faecalibacterium* | E_R_PRCG | -0.0235 | 0.8550 | 64 |
| *Lachnospiraceae incertae sedis* | B_R_Nacc | 0.3308 | 0.0081 | 64 |
| *Lachnospiraceae incertae sedis* | B_L_SupFG | 0.3230 | 0.0098 | 64 |
| *Lachnospiraceae incertae sedis* | B_R_SbCG_S | 0.3148 | 0.0120 | 64 |
| *Lachnospiraceae incertae sedis* | B_R_SupFG | 0.3132 | 0.0124 | 64 |
| *Lachnospiraceae incertae sedis* | B_L_SbCG_S | 0.2958 | 0.0186 | 64 |
| *Lachnospiraceae incertae sedis* | B_L_InfCirInS | -0.2891 | 0.0215 | 64 |
| *Lachnospiraceae incertae sedis* | B_R_PosLS | -0.2643 | 0.0363 | 64 |
| *Lachnospiraceae incertae sedis* | B_L_InfPrCS | -0.2531 | 0.0454 | 64 |
| *Lachnospiraceae incertae sedis* | B_R_PosCS | -0.2282 | 0.0720 | 64 |
| *Lachnospiraceae incertae sedis* | B_R_SupFS | -0.1624 | 0.2034 | 64 |
| *Lachnospiraceae incertae sedis* | E_R_PosCS | -0.1539 | 0.2285 | 64 |
| *Lachnospiraceae incertae sedis* | B_L_PRCG | -0.1447 | 0.2580 | 64 |
| *Lachnospiraceae incertae sedis* | B_L_PosCG | -0.1289 | 0.3139 | 64 |
| *Lachnospiraceae incertae sedis* | B_L_PosCS | -0.1111 | 0.3859 | 64 |
| *Lachnospiraceae incertae sedis* | B_L_CS | -0.0746 | 0.5611 | 64 |
| *Lachnospiraceae incertae sedis* | B_R_CS | -0.0524 | 0.6832 | 64 |
| *Lachnospiraceae incertae sedis* | E_L_PosCS | -0.0301 | 0.8151 | 64 |
| *Lachnospiraceae incertae sedis* | B_R_SupFG | 0.0260 | 0.8397 | 64 |
| *Oscillibacter* | B_L_CaN | -0.3019 | 0.0162 | 64 |
| *Oscillibacter* | B_L_LoInG_CInS | 0.2906 | 0.0209 | 64 |
| *Oscillibacter* | B_R_PosLS | 0.2766 | 0.0282 | 64 |
| *Oscillibacter* | B_R_InfPrCS | 0.2675 | 0.0340 | 64 |
| *Oscillibacter* | B_R_SupCirInS | 0.2502 | 0.0480 | 64 |
| *Oscillibacter* | E_L_InfCirInS | 0.0385 | 0.7644 | 64 |
| *Roseburia* | B_R_LoInG_CInS | 0.4005 | 0.0011 | 64 |
| *Roseburia* | B_R_SupCirInS | -0.3669 | 0.0031 | 64 |
| *Roseburia* | B_L_PosLS | -0.2906 | 0.0209 | 64 |
| *Roseburia* | B_R_LoInG_CInS | 0.2802 | 0.0261 | 64 |
| *Roseburia* | E_R_LoInG_CInS | 0.2640 | 0.0365 | 64 |
| *Roseburia* | E_L_Pal | -0.2503 | 0.0479 | 64 |
| *Roseburia* | B_L_CaN | -0.0550 | 0.6683 | 64 |
| *Roseburia* | B_R_Pu | 0.0260 | 0.8399 | 64 |
| *Roseburia* | B_R_SupFS | -0.0005 | 0.9967 | 64 |
|  |  |  |  |  |
| **Microbial Genus** | **Clinical Measure** | ***r*** | ***p*** | ***df*** |
| *Clostridium IV* | Rectal Discomfort Intensity | 0.3366 | 0.0091 | 60 |
| *Clostridium XlVa* | Lactulose - Pain | 0.3226 | 0.0099 | 64 |
| *Clostridium XlVa* | Rectal Pain Threshold | 0.0956 | 0.4673 | 64 |
| *Clostridium XlVb* | Lactulose - Pain | 0.3085 | 0.0139 | 64 |
| *Coprococcus* | Lactulose - Pain | -0.1754 | 0.1691 | 64 |
| *Coprococcus* | Lactulose - Discomfort | -0.0246 | 0.8482 | 64 |
| *Oscillibacter* | First Rectal Sensation | -0.3794 | 0.0028 | 61 |
| *Roseburia* | Lactulose - Discomfort | 0.2770 | 0.0280 | 64 |
|  |  |  |  |  |
| **Clinical Measure** | **Functional Connectivity** | ***r*** | ***p*** | ***df*** |
| First Rectal Sensation | B_L_InfCirInS | -0.3087 | 0.0164 | 61 |
| First Rectal Sensation | B_R_InfCirInS | -0.3076 | 0.0168 | 61 |
| First Rectal Sensation | B_L_Pal | -0.3054 | 0.0177 | 61 |
| First Rectal Sensation | B_R_LoInG_CInS | -0.2958 | 0.0217 | 61 |
| First Rectal Sensation | B_L_PRCG | 0.2954 | 0.0220 | 61 |
| First Rectal Sensation | E_L_InfPrCS | 0.2865 | 0.0265 | 61 |
| First Rectal Sensation | B_R_SupFS | 0.1187 | 0.3665 | 61 |
| First Rectal Sensation | B_L_SupFS | -0.0208 | 0.8748 | 61 |
| IBS-SSS | B_L_Tha | 0.3279 | 0.0087 | 64 |
| IBS-SSS | B_R_Tha | 0.2899 | 0.0212 | 64 |
| IBS-SSS | E_R_Tha | 0.2854 | 0.0234 | 64 |
| IBS-SSS | B_L_SupCirInS | -0.2748 | 0.0293 | 64 |
| IBS-SSS | B_L_PosCG | 0.2506 | 0.0476 | 64 |
| IBS-SSS | B_L_SupPrCs | 0.1878 | 0.1405 | 64 |
| IBS-SSS | E_L_SupFS | 0.1286 | 0.3150 | 64 |
| IBS-SSS | E_R_CS | -0.0978 | 0.4457 | 64 |
| IBS-SSS | E_L_CS | -0.0724 | 0.5727 | 64 |
| IBS-SSS | E_L_PosCG | 0.0328 | 0.7988 | 64 |
| Lactulose - Discomfort | B_L_PRCG | -0.3616 | 0.0036 | 64 |
| Lactulose - Discomfort | B_L_PosLS | -0.3436 | 0.0058 | 64 |
| Lactulose - Discomfort | B_R_PosLS | -0.3327 | 0.0077 | 64 |
| Lactulose - Discomfort | B_R_PosCG | -0.3236 | 0.0097 | 64 |
| Lactulose - Discomfort | E_L_PRCG | -0.3156 | 0.0117 | 64 |
| Lactulose - Discomfort | B_L_InfCirInS | 0.3030 | 0.0158 | 64 |
| Lactulose - Discomfort | B_L_CS | -0.3001 | 0.0169 | 64 |
| Lactulose - Discomfort | E_L_Tha | 0.2999 | 0.0169 | 64 |
| Lactulose - Discomfort | B_L_PosCG | -0.2873 | 0.0224 | 64 |
| Lactulose - Discomfort | B_L_Tha | 0.2866 | 0.0228 | 64 |
| Lactulose - Discomfort | E_R_Tha | 0.2853 | 0.0234 | 64 |
| Lactulose - Discomfort | B_R_PRCG | -0.2820 | 0.0251 | 64 |
| Lactulose - Discomfort | B_L_SupCirInS | -0.2751 | 0.0291 | 64 |
| Lactulose - Discomfort | B_R_CS | -0.2728 | 0.0305 | 64 |
| Lactulose - Discomfort | B_L_SbCG_S | -0.2650 | 0.0358 | 64 |
| Lactulose - Discomfort | B_L_PosLS | -0.2649 | 0.0359 | 64 |
| Lactulose - Discomfort | B_R_SbCG_S | -0.2510 | 0.0472 | 64 |
| Lactulose - Discomfort | B_L_PosCS | -0.2488 | 0.0493 | 64 |
| Lactulose - Discomfort | B_L_LoInG_CInS | -0.2481 | 0.0499 | 64 |
| Lactulose - Discomfort | B_R_LoInG_CInS | -0.1926 | 0.1304 | 64 |
| Lactulose - Discomfort | B_L_PosCG | 0.1650 | 0.1963 | 64 |
| Lactulose - Discomfort | E_R_SbCG_S | -0.1227 | 0.3382 | 64 |
| Lactulose - Discomfort | E_L_SupFS | 0.0156 | 0.9033 | 64 |
| Lactulose - Pain | B_L_Tha | 0.3281 | 0.0087 | 64 |
| Lactulose - Pain | E_L_PRCG | -0.2799 | 0.0263 | 64 |
| Lactulose - Pain | B_R_SupFS | -0.2573 | 0.0418 | 64 |
| Lactulose - Pain | E_R_Nacc | -0.1041 | 0.4169 | 64 |
| Lactulose - Pain | B_R_PosLS | -0.0864 | 0.5007 | 64 |
| Lactulose - Pain | E_L_Nacc | -0.0566 | 0.6594 | 64 |
| Lactulose - Pain | B_R_SbCG_S | -0.0516 | 0.6877 | 64 |
| Lactulose - Pain | B_L_SbCG_S | -0.0501 | 0.6967 | 64 |
| Lactulose - Pain | B_L_PosLS | -0.0485 | 0.7058 | 64 |
| Lactulose - Pain | E_R_CaN | 0.0432 | 0.7368 | 64 |
| Lactulose - Pain | E_L_CaN | 0.0312 | 0.8083 | 64 |
| Lactulose - Pain | B_L_CaN | 0.0263 | 0.8380 | 64 |
| OATT | B_L_SupPrCs | -0.2727 | 0.0306 | 64 |
| OATT | B_R_CS | 0.2573 | 0.0417 | 64 |
| OATT | B_L_Pu | -0.2342 | 0.0646 | 64 |
| OATT | B_L_SupCirInS | 0.2331 | 0.0660 | 64 |
| OATT | B_L_Nacc | 0.1123 | 0.3808 | 64 |
| OATT | B_R_SbCG_S | -0.0803 | 0.5313 | 64 |
| OATT | B_L_PosLS | -0.0244 | 0.8495 | 64 |
| OATT | B_R_Pu | 0.0126 | 0.9219 | 64 |
| Rectal Discomfort Intensity | B_R_SupCirInS | -0.3180 | 0.0141 | 60 |
| Rectal Discomfort Intensity | B_R_Tha | 0.2801 | 0.0316 | 60 |
| Rectal Discomfort Intensity | E_L_SupCirInS | -0.2734 | 0.0362 | 60 |
| Rectal Discomfort Intensity | E_R_SupCirInS | -0.2708 | 0.0381 | 60 |
| Rectal Discomfort Intensity | B_R_CaN | -0.1677 | 0.2043 | 60 |
| Rectal Discomfort Intensity | E_L_InfPrCS | 0.1597 | 0.2271 | 60 |
| Rectal Discomfort Intensity | B_L_PosCS | -0.0305 | 0.8188 | 60 |
| Rectal Discomfort Threshold | B_L_Pu | 0.1577 | 0.2289 | 60 |
| Rectal Discomfort Threshold | B_R_CaN | 0.1480 | 0.2592 | 60 |
| Rectal Discomfort Threshold | B_L_SupCirInS | -0.0182 | 0.8900 | 60 |
| Rectal Pain Intensity | B_L_SupCirInS | 0.4544 | 0.0003 | 60 |
| Rectal Pain Intensity | B_R_Tha | 0.3050 | 0.0188 | 60 |
| Rectal Pain Intensity | E_R_PosLS | -0.3040 | 0.0193 | 60 |
| Rectal Pain Intensity | B_R_SbCG_S | 0.2802 | 0.0316 | 60 |
| Rectal Pain Intensity | B_L_Tha | 0.2789 | 0.0324 | 60 |
| Rectal Pain Intensity | B_R_PosLS | -0.2786 | 0.0326 | 60 |
| Rectal Pain Intensity | B_R_InfPrCS | -0.2649 | 0.0426 | 60 |
| Rectal Pain Intensity | B_R_PRCG | -0.2623 | 0.0448 | 60 |
| Rectal Pain Intensity | B_L_SupFS | 0.2606 | 0.0462 | 60 |
| Rectal Pain Intensity | B_R_PRCG | -0.2587 | 0.0479 | 60 |
| Rectal Pain Intensity | E_L_SupFG | 0.2281 | 0.0823 | 60 |
| Rectal Pain Intensity | B_L_PosCS | 0.1382 | 0.2965 | 60 |
| Rectal Pain Intensity | B_R_CaN | 0.0928 | 0.4844 | 60 |
| Rectal Pain Intensity | B_L_Nacc | -0.0688 | 0.6046 | 60 |
| Rectal Pain Threshold | E_R_CS | 0.2864 | 0.0265 | 61 |
| Rectal Pain Threshold | B_L_SupFS | -0.2766 | 0.0324 | 61 |
| Rectal Pain Threshold | B_L_Pu | 0.2513 | 0.0528 | 61 |
| Rectal Pain Threshold | E_L_CS | 0.2431 | 0.0613 | 61 |
| Rectal Pain Threshold | B_R_LoInG_CInS | -0.1947 | 0.1361 | 61 |
| Rectal Pain Threshold | B_R_CS | 0.1813 | 0.1658 | 61 |
| Rectal Pain Threshold | B_L_CS | 0.1552 | 0.2365 | 61 |
| Rectal Pain Threshold | E_R_PosCG | 0.1517 | 0.2472 | 61 |
| Rectal Pain Threshold | E_L_PosCG | 0.1404 | 0.2846 | 61 |
| Rectal Pain Threshold | B_L_PRCG | 0.1286 | 0.3273 | 61 |
| Rectal Pain Threshold | B_R_PosCG | 0.0988 | 0.4527 | 61 |
| Rectal Pain Threshold | B_L_PosCG | 0.0872 | 0.5078 | 61 |
| Rectal Pain Threshold | E_R_CaN | 0.0845 | 0.5211 | 61 |
| Rectal Pain Threshold | B_R_PosCS | 0.0713 | 0.5883 | 61 |
| Rectal Pain Threshold | E_L_Tha | -0.0708 | 0.5906 | 61 |
| Rectal Pain Threshold | B_L_SbCG_S | 0.0655 | 0.6192 | 61 |
| Rectal Pain Threshold | E_L_Pu | 0.0571 | 0.6645 | 61 |
| Rectal Pain Threshold | B_R_SbCG_S | 0.0451 | 0.7324 | 61 |
| Rectal Pain Threshold | B_L_PosCS | 0.0417 | 0.7519 | 61 |
| Rectal Pain Threshold | E_R_PosCS | 0.0372 | 0.7779 | 61 |
| Rectal Pain Threshold | B_R_PosLS | 0.0281 | 0.8311 | 61 |
| Rectal Pain Threshold | B_L_Nacc | 0.0236 | 0.8579 | 61 |
| Rectal Pain Threshold | E_R_Tha | -0.0039 | 0.9766 | 61 |
