# Supplementary material for: Evidence for an association of gut microbial Clostridia with brain functional connectivity and gastrointestinal sensorimotor function in patients with irritable bowel syndrome, based on tripartite network analysis
Source: Microbiome. 2019 Mar 21;7:45. doi: 10.1186/s40168-019-0656-z (PMC6429755; doi:10.1186/s40168-019-0656-z)
Supplement: Supplementary file 3 — Table S3. Difference Network Z Tests. This table shows all of the Z tests that define the difference network. Functional connectivity of regions of interest are presented in the format: X_Y_Z, where X indicates a connectivity measure (B, Betweenness centrality; E, Eigenvector centrality; S, Degree strength), Y indicates lateralization (L, Left; R, Right), and Z indicates a region of interest (see Table 1). Abbreviations: First Rectal Sensation, first sensation threshold during balloon distension; Rectal Discomfort Threshold, discomfort threshold during balloon distension; IBS-SS, Irritable Bowel Syndrome - Scoring System scores; Lactulose - Discomfort, discomfort during nutrient and lactulose challenge test; Lactulose - Pain, pain during lactulose challenge test; OATT, oroanal transit time; Rectal Pain Threshold, pain threshold during balloon distension; Rectal Discomfort Intensity, visual analogue scale rating of discomfort during 24 mmHg distension; Rectal Pain Intensity, visual analogue scale rating of pain during 24 mmHg distension. DOCX 156 kb) [file 40168_2019_656_MOESM3_ESM.docx]

**Table S3. Difference Network Z Tests**

| **Microbial Genus** | **Functional Connectivity** | ***z*** | ***p*** | ***n(IBS)*** | ***n(HC)*** |
| --- | --- | --- | --- | --- | --- |
| *Blautia* | E_R_LoInG_CInS | -2.75 | 0.0060 | 65 | 21 |
| *Blautia* | B_L_SupFS | 2.52 | 0.0117 | 65 | 21 |
| *Blautia* | B_L_SupPrCs | -2.46 | 0.0139 | 65 | 21 |
| *Blautia* | B_R_InfPrCS | -2.4 | 0.0164 | 65 | 21 |
| *Blautia* | B_L_InfPrCS | 1.91 | 0.0561 | 65 | 21 |
| *Blautia* | B_L_InfPrCS | -1.83 | 0.0673 | 65 | 21 |
| *Blautia* | B_R_InfCirInS | -1.48 | 0.1389 | 65 | 21 |
| *Blautia* | E_R_InfCirInS | 1.25 | 0.2113 | 65 | 21 |
| *Blautia* | B_R_SupCirInS | 0.91 | 0.3628 | 65 | 21 |
| *Blautia* | B_R_SupFS | -0.41 | 0.6818 | 65 | 21 |
| *Blautia* | B_R_LoInG_CInS | -0.32 | 0.7490 | 65 | 21 |
| *Clostridium IV* | B_L_SupFG | -3.11 | 0.0019 | 65 | 21 |
| *Clostridium IV* | B_L_PRCG | -2.01 | 0.0444 | 65 | 21 |
| *Clostridium IV* | B_L_InfCirInS | -1.88 | 0.0601 | 65 | 21 |
| *Clostridium IV* | B_R_InfCirInS | -1.88 | 0.0601 | 65 | 21 |
| *Clostridium IV* | B_L_SupPrCs | 1.41 | 0.1585 | 65 | 21 |
| *Clostridium IV* | B_R_Tha | 0.65 | 0.5157 | 65 | 21 |
| *Clostridium IV* | B_R_PosCG | 0.28 | 0.7795 | 65 | 21 |
| *Clostridium XlVa* | B_L_PosCG | -3.38 | 0.0007 | 65 | 21 |
| *Clostridium XlVa* | B_R_SbCG_S | 2.81 | 0.0050 | 65 | 21 |
| *Clostridium XlVa* | E_L_Pu | 2.73 | 0.0063 | 65 | 21 |
| *Clostridium XlVa* | B_L_Nacc | -2.71 | 0.0067 | 65 | 21 |
| *Clostridium XlVa* | B_L_Pu | -2.68 | 0.0074 | 65 | 21 |
| *Clostridium XlVa* | B_R_PosCG | -2.64 | 0.0083 | 65 | 21 |
| *Clostridium XlVa* | B_L_Tha | -2.37 | 0.0178 | 65 | 21 |
| *Clostridium XlVa* | E_R_Pu | 2.22 | 0.0264 | 65 | 21 |
| *Clostridium XlVa* | B_L_CS | -1.97 | 0.0488 | 65 | 21 |
| *Clostridium XlVa* | B_L_PosCS | 1.86 | 0.0629 | 65 | 21 |
| *Clostridium XlVa* | B_L_SbCG_S | 1.65 | 0.0989 | 65 | 21 |
| *Clostridium XlVa* | B_L_PosLS | 1.61 | 0.1074 | 65 | 21 |
| *Clostridium XlVa* | E_L_CaN | 1.57 | 0.1164 | 65 | 21 |
| *Clostridium XlVa* | B_L_PRCG | 1.8 | 0.1389 | 65 | 21 |
| *Clostridium XlVa* | B_R_CS | -1.21 | 0.2263 | 65 | 21 |
| *Clostridium XlVa* | B_R_PRCG | 1.21 | 0.2263 | 65 | 21 |
| *Clostridium XlVa* | B_L_InfCirInS | -0.03 | 0.9761 | 65 | 21 |
| *Clostridium XlVb* | E_L_Tha | -2.51 | 0.0121 | 65 | 21 |
| *Clostridium XlVb* | B_L_Tha | 2.32 | 0.0203 | 65 | 21 |
| *Clostridium XlVb* | E_L_SbCG_S | -2.02 | 0.0434 | 65 | 21 |
| *Clostridium XlVb* | B_R_InfCirInS | -1.87 | 0.0615 | 65 | 21 |
| *Clostridium XlVb* | B_R_LoInG_CInS | 1.8 | 0.0719 | 65 | 21 |
| *Clostridium XlVb* | E_L_SupFS | -1.79 | 0.0735 | 65 | 21 |
| *Clostridium XlVb* | B_R_PosLS | -1.65 | 0.0989 | 65 | 21 |
| *Clostridium XlVb* | B_L_Tha | -1.5 | 0.1336 | 65 | 21 |
| *Clostridium XlVb* | E_R_PRCG | 1.47 | 0.1416 | 65 | 21 |
| *Clostridium XlVb* | E_L_Nacc | 1.13 | 0.2585 | 65 | 21 |
| *Clostridium XlVb* | B_R_PRCG | 0.95 | 0.3421 | 65 | 21 |
| *Clostridium XlVb* | B_R_Pu | -0.67 | 0.5029 | 65 | 21 |
| *Clostridium XlVb* | B_L_SupPrCs | -0.48 | 0.6312 | 65 | 21 |
| *Clostridium XlVb* | B_R_Tha | 0.31 | 0.7566 | 65 | 21 |
| *Clostridium XlVb* | E_R_CaN | 0.23 | 0.8181 | 65 | 21 |
| *Clostridium XlVb* | E_R_CS | 0.18 | 0.8572 | 65 | 21 |
| *Clostridium XlVb* | E_R_Pu | -0.09 | 0.9283 | 65 | 21 |
| *Coprococcus* | B_R_CaN | 3.76 | 0.0002 | 65 | 21 |
| *Coprococcus* | B_L_CaN | -2.55 | 0.0108 | 65 | 21 |
| *Coprococcus* | E_L_SupFS | -2.48 | 0.0131 | 65 | 21 |
| *Coprococcus* | B_L_SbCG_S | -2.11 | 0.0349 | 65 | 21 |
| *Coprococcus* | B_L_SupFS | -1.8 | 0.0719 | 65 | 21 |
| *Coprococcus* | B_L_PosLS | -1.52 | 0.1285 | 65 | 21 |
| *Coprococcus* | E_R_CaN | 1.44 | 0.1499 | 65 | 21 |
| *Coprococcus* | B_R_PosCG | 1.24 | 0.2150 | 65 | 21 |
| *Coprococcus* | B_R_InfCirInS | -1.03 | 0.3030 | 65 | 21 |
| *Coprococcus* | B_R_SbCG_S | 0.91 | 0.3628 | 65 | 21 |
| *Coprococcus* | E_L_CaN | 0.43 | 0.6672 | 65 | 21 |
| *Coprococcus* | E_L_InfPrCS | -0.42 | 0.6745 | 65 | 21 |
| *Faecalibacterium* | E_R_PRCG | 1.11 | 0.2670 | 65 | 21 |
| *Faecalibacterium* | B_R_PosLS | -1.65 | 0.9890 | 65 | 21 |
| *Lachnospiraceae incertae sedis* | B_L_PosCG | 3.26 | 0.0011 | 65 | 21 |
| *Lachnospiraceae incertae sedis* | E_R_PosCS | -3.13 | 0.0017 | 65 | 21 |
| *Lachnospiraceae incertae sedis* | B_L_InfPrCS | 2.56 | 0.0105 | 65 | 21 |
| *Lachnospiraceae incertae sedis* | E_L_PosCS | 2.46 | 0.0139 | 65 | 21 |
| *Lachnospiraceae incertae sedis* | B_R_CS | 2.27 | 0.0232 | 65 | 21 |
| *Lachnospiraceae incertae sedis* | B_L_CS | -2.05 | 0.0404 | 65 | 21 |
| *Lachnospiraceae incertae sedis* | B_R_Nacc | 1.99 | 0.0466 | 65 | 21 |
| *Lachnospiraceae incertae sedis* | B_R_PosCS | 1.98 | 0.0477 | 65 | 21 |
| *Lachnospiraceae incertae sedis* | B_L_SbCG_S | 1.8 | 0.0719 | 65 | 21 |
| *Lachnospiraceae incertae sedis* | B_L_InfCirInS | 1.72 | 0.0854 | 65 | 21 |
| *Lachnospiraceae incertae sedis* | B_L_PRCG | 1.27 | 0.2041 | 65 | 21 |
| *Lachnospiraceae incertae sedis* | B_R_SupFG | 1.07 | 0.2846 | 65 | 21 |
| *Lachnospiraceae incertae sedis* | B_L_PosCS | -0.93 | 0.3524 | 65 | 21 |
| *Lachnospiraceae incertae sedis* | B_R_SupFG | -0.9 | 0.3681 | 65 | 21 |
| *Lachnospiraceae incertae sedis* | B_L_SupFG | -0.88 | 0.3789 | 65 | 21 |
| *Lachnospiraceae incertae sedis* | B_R_SbCG_S | 0.57 | 0.5687 | 65 | 21 |
| *Lachnospiraceae incertae sedis* | B_R_PosLS | 0.31 | 0.7566 | 65 | 21 |
| *Lachnospiraceae incertae sedis* | B_R_SupFS | 0.03 | 0.9761 | 65 | 21 |
| *Oscillibacter* | B_L_CaN | 2.47 | 0.0135 | 65 | 21 |
| *Oscillibacter* | B_L_LoInG_CInS | 2.03 | 0.0424 | 65 | 21 |
| *Oscillibacter* | B_R_PosLS | -1.87 | 0.0615 | 65 | 21 |
| *Oscillibacter* | B_R_InfPrCS | -1.81 | 0.0703 | 65 | 21 |
| *Oscillibacter* | E_L_InfCirInS | 1.68 | 0.0930 | 65 | 21 |
| *Oscillibacter* | B_R_SupCirInS | 0.16 | 0.8729 | 65 | 21 |
| *Roseburia* | E_L_Pal | -2.59 | 0.0096 | 65 | 21 |
| *Roseburia* | E_R_LoInG_CInS | 2.23 | 0.0257 | 65 | 21 |
| *Roseburia* | B_L_PosLS | -2.15 | 0.0316 | 65 | 21 |
| *Roseburia* | B_L_CaN | -2.08 | 0.0375 | 65 | 21 |
| *Roseburia* | B_R_LoInG_CInS | 1.67 | 0.0949 | 65 | 21 |
| *Roseburia* | B_R_Pu | -1.32 | 0.1868 | 65 | 21 |
| *Roseburia* | B_R_LoInG_CInS | 0.49 | 0.6241 | 65 | 21 |
| *Roseburia* | B_R_SupFS | 0.22 | 0.8259 | 65 | 21 |
| *Roseburia* | B_R_SupCirInS | 0.16 | 0.8729 | 65 | 21 |
|  |  |  |  |  |  |
| **Microbial Genus** | **Clinical Measure** | ***z*** | ***p*** | ***n(IBS)*** | ***n(HC)*** |
| *Clostridium IV* | Rectal Discomfort Intensity | -0.08 | 0.9362 | 65 | 21 |
| *Clostridium XlVa* | Rectal Pain Threshold | -0.98 | 0.3271 | 62 | 21 |
| *Clostridium XlVa* | Lactulose - Pain | -0.06 | 0.9522 | 65 | 21 |
| *Clostridium XlVb* | Lactulose - Pain | -2.81 | 0.005 | 65 | 21 |
| *Coprococcus* | Lactulose - Discomfort | -1.6 | 0.1096 | 65 | 21 |
| *Coprococcus* | Lactulose - Pain | 1.41 | 0.1585 | 65 | 21 |
| *Oscillibacter* | First Rectal Sensation | 1.27 | 0.2041 | 61 | 21 |
| *Roseburia* | Lactulose - Discomfort | 2.02 | 0.0434 | 65 | 21 |
|  |  |  |  |  |  |
| **Clinical Measure** | **Functional Connectivity** | ***z*** | ***p*** | ***n(IBS)*** | ***n(HC)*** |
| First Rectal Sensation | B_L_PRCG | 2.44 | 0.0147 | 62 | 21 |
| First Rectal Sensation | B_R_InfCirInS | -2.37 | 0.0178 | 62 | 21 |
| First Rectal Sensation | B_L_InfCirInS | -2.17 | 0.03 | 62 | 21 |
| First Rectal Sensation | E_L_InfPrCS | 2.15 | 0.0316 | 62 | 21 |
| First Rectal Sensation | B_R_LoInG_CInS | -2.09 | 0.0366 | 62 | 21 |
| First Rectal Sensation | B_L_Pal | 1.87 | 0.0615 | 65 | 21 |
| First Rectal Sensation | B_L_SupFS | 1.86 | 0.0629 | 65 | 21 |
| First Rectal Sensation | B_R_SupFS | 0.67 | 0.5029 | 65 | 21 |
| IBS-SSS | B_L_PosCG | -2.76 | 0.0076 | 65 | 21 |
| IBS-SSS | B_L_Tha | -2.6 | 0.0093 | 65 | 21 |
| IBS-SSS | E_R_Tha | -2.4 | 0.0164 | 65 | 21 |
| IBS-SSS | E_L_PosCG | 2.32 | 0.0203 | 65 | 21 |
| IBS-SSS | E_L_SupFS | 2.02 | 0.0217 | 65 | 21 |
| IBS-SSS | B_L_SupCirInS | 2.23 | 0.0257 | 65 | 21 |
| IBS-SSS | B_L_SupPrCs | -2.14 | 0.0324 | 65 | 21 |
| IBS-SSS | E_R_CS | 1.98 | 0.0477 | 65 | 21 |
| IBS-SSS | E_L_CS | 0.59 | 0.5552 | 65 | 21 |
| IBS-SSS | B_R_Tha | -0.4 | 0.6892 | 65 | 21 |
| Lactulose - Discomfort | B_L_Tha | -3.43 | 0.0006 | 65 | 21 |
| Lactulose - Discomfort | B_L_CS | -2.59 | 0.0096 | 65 | 21 |
| Lactulose - Discomfort | B_L_PosCS | -2.31 | 0.0209 | 65 | 21 |
| Lactulose - Discomfort | E_L_SupFS | -2.04 | 0.0414 | 65 | 21 |
| Lactulose - Discomfort | B_R_PosCG | -1.97 | 0.0488 | 65 | 21 |
| Lactulose - Discomfort | B_R_PRCG | 1.93 | 0.0536 | 65 | 21 |
| Lactulose - Discomfort | E_L_Tha | -1.9 | 0.0574 | 65 | 21 |
| Lactulose - Discomfort | B_R_LoInG_CInS | -1.81 | 0.0703 | 65 | 21 |
| Lactulose - Discomfort | B_L_PosLS | -1.78 | 0.0751 | 65 | 21 |
| Lactulose - Discomfort | B_R_CS | -1.77 | 0.0767 | 65 | 21 |
| Lactulose - Discomfort | B_R_PosLS | -1.74 | 0.0819 | 65 | 21 |
| Lactulose - Discomfort | E_L_PRCG | -1.71 | 0.0873 | 65 | 21 |
| Lactulose - Discomfort | B_L_InfCirInS | 1.71 | 0.0873 | 65 | 21 |
| Lactulose - Discomfort | E_R_Tha | 1.7 | 0.0891 | 65 | 21 |
| Lactulose - Discomfort | B_L_LoInG_CInS | 1.64 | 0.101 | 65 | 21 |
| Lactulose - Discomfort | B_L_SbCG_S | -1.46 | 0.1443 | 65 | 21 |
| Lactulose - Discomfort | E_R_SbCG_S | 1.18 | 0.238 | 65 | 21 |
| Lactulose - Discomfort | B_L_SupCirInS | -0.9 | 0.3681 | 65 | 21 |
| Lactulose - Discomfort | B_L_PRCG | 0.71 | 0.4777 | 65 | 21 |
| Lactulose - Discomfort | B_L_PosLS | -0.51 | 0.6101 | 65 | 21 |
| Lactulose - Discomfort | B_L_PosCG | 0.5 | 0.6171 | 65 | 21 |
| Lactulose - Discomfort | B_L_PosCG | 0.11 | 0.9124 | 65 | 21 |
| Lactulose - Discomfort | B_R_SbCG_S | 0.11 | 0.9124 | 65 | 21 |
| Lactulose - Pain | B_L_Tha | -2.41 | 0.016 | 65 | 21 |
| Lactulose - Pain | B_L_CaN | -2.31 | 0.0209 | 65 | 21 |
| Lactulose - Pain | B_L_SbCG_S | 2.2 | 0.0278 | 65 | 21 |
| Lactulose - Pain | B_R_SbCG_S | -2.2 | 0.0278 | 65 | 21 |
| Lactulose - Pain | E_L_Nacc | -2.06 | 0.0394 | 65 | 21 |
| Lactulose - Pain | E_L_PRCG | -1.99 | 0.0466 | 65 | 21 |
| Lactulose - Pain | E_R_Nacc | -1.88 | 0.0601 | 65 | 21 |
| Lactulose - Pain | B_R_PosLS | -1.5 | 0.1336 | 65 | 21 |
| Lactulose - Pain | E_R_CaN | -1.18 | 0.238 | 65 | 21 |
| Lactulose - Pain | B_R_SupFS | 1.14 | 0.2543 | 65 | 21 |
| Lactulose - Pain | E_L_CaN | 1.02 | 0.3077 | 65 | 21 |
| Lactulose - Pain | B_L_PosLS | -0.1 | 0.9203 | 65 | 21 |
| OATT | B_L_Nacc | 2.7 | 0.0069 | 65 | 21 |
| OATT | B_R_CS | 2.25 | 0.0244 | 65 | 21 |
| OATT | B_L_SupPrCs | 2.2 | 0.0278 | 65 | 21 |
| OATT | B_L_SupCirInS | 1.78 | 0.0751 | 65 | 21 |
| OATT | B_R_Pu | 0.8 | 0.4237 | 65 | 21 |
| OATT | B_R_SbCG_S | -0.8 | 0.4237 | 61 | 21 |
| OATT | B_L_Pu | 0.77 | 0.4413 | 61 | 21 |
| OATT | B_L_PosLS | -0.63 | 0.5287 | 61 | 21 |
| Rectal Discomfort Intensity | E_L_InfPrCS | 2.46 | 0.0139 | 62 | 21 |
| Rectal Discomfort Intensity | E_L_SupCirInS | -2.29 | 0.022 | 62 | 21 |
| Rectal Discomfort Intensity | E_R_SupCirInS | 1.91 | 0.0561 | 62 | 21 |
| Rectal Discomfort Intensity | B_R_CaN | -1.77 | 0.0767 | 62 | 21 |
| Rectal Discomfort Intensity | B_R_SupCirInS | 1.72 | 0.0854 | 62 | 21 |
| Rectal Discomfort Intensity | B_L_PosCS | -1.6 | 0.1096 | 62 | 21 |
| Rectal Discomfort Intensity | B_R_Tha | 1.56 | 0.1188 | 62 | 21 |
| Rectal Discomfort Threshold | B_R_CaN | -3.36 | 0.0008 | 62 | 21 |
| Rectal Discomfort Threshold | B_L_SupCirInS | -2.71 | 0.0067 | 62 | 21 |
| Rectal Discomfort Threshold | B_L_Pu | 2.17 | 0.03 | 62 | 21 |
| Rectal Pain Intensity | B_R_PosLS | -3.39 | 0.0007 | 62 | 21 |
| Rectal Pain Intensity | B_L_SupCirInS | -2.99 | 0.0028 | 62 | 21 |
| Rectal Pain Intensity | B_R_CaN | -2.94 | 0.0033 | 62 | 21 |
| Rectal Pain Intensity | B_L_Nacc | 2.68 | 0.0074 | 62 | 21 |
| Rectal Pain Intensity | B_L_Tha | -2.1 | 0.0357 | 62 | 21 |
| Rectal Pain Intensity | B_R_InfPrCS | -1.78 | 0.0751 | 62 | 21 |
| Rectal Pain Intensity | E_L_SupFG | -1.67 | 0.0949 | 62 | 21 |
| Rectal Pain Intensity | B_L_SupFS | -1.33 | 0.1835 | 62 | 21 |
| Rectal Pain Intensity | E_R_PosLS | 1.24 | 0.215 | 62 | 21 |
| Rectal Pain Intensity | B_R_Tha | 1.23 | 0.2187 | 62 | 21 |
| Rectal Pain Intensity | B_R_PRCG | -1.07 | 0.2846 | 62 | 21 |
| Rectal Pain Intensity | B_L_PosCS | -0.42 | 0.6745 | 62 | 21 |
| Rectal Pain Intensity | B_R_PRCG | 0.4 | 0.6892 | 62 | 21 |
| Rectal Pain Intensity | B_R_SbCG_S | 0.4 | 0.6892 | 62 | 21 |
| Rectal Pain Threshold | E_R_CS | 3.06 | 0.0022 | 61 | 21 |
| Rectal Pain Threshold | B_L_Pu | 2.91 | 0.0036 | 61 | 21 |
| Rectal Pain Threshold | E_L_Pu | 2.86 | 0.0042 | 61 | 21 |
| Rectal Pain Threshold | B_L_SbCG_S | 2.6 | 0.0093 | 61 | 21 |
| Rectal Pain Threshold | E_L_PosCG | 2.58 | 0.0099 | 61 | 21 |
| Rectal Pain Threshold | B_L_PosCG | 2.28 | 0.0226 | 61 | 21 |
| Rectal Pain Threshold | B_R_PosLS | -2.27 | 0.0232 | 61 | 21 |
| Rectal Pain Threshold | E_L_Tha | -2.18 | 0.0293 | 61 | 21 |
| Rectal Pain Threshold | E_R_Tha | 2.14 | 0.0324 | 61 | 21 |
| Rectal Pain Threshold | B_R_CS | -2.1 | 0.0357 | 61 | 21 |
| Rectal Pain Threshold | B_L_Nacc | 1.89 | 0.0588 | 61 | 21 |
| Rectal Pain Threshold | B_R_LoInG_CInS | -1.87 | 0.0615 | 61 | 21 |
| Rectal Pain Threshold | B_R_PosCG | 1.8 | 0.0719 | 61 | 21 |
| Rectal Pain Threshold | B_L_PosCS | -1.55 | 0.1211 | 61 | 21 |
| Rectal Pain Threshold | B_L_SupFS | 1.48 | 0.1389 | 61 | 21 |
| Rectal Pain Threshold | E_R_PosCG | 1.04 | 0.2983 | 61 | 21 |
| Rectal Pain Threshold | B_L_PRCG | 0.9 | 0.3681 | 61 | 21 |
| Rectal Pain Threshold | E_R_PosCS | 0.79 | 0.4295 | 61 | 21 |
| Rectal Pain Threshold | B_L_CS | 0.7 | 0.4839 | 61 | 21 |
| Rectal Pain Threshold | E_R_CaN | 0.56 | 0.5755 | 61 | 21 |
| Rectal Pain Threshold | E_L_CS | 0.51 | 0.6101 | 61 | 21 |
| Rectal Pain Threshold | B_R_PosCS | 0.35 | 0.7263 | 62 | 21 |
| Rectal Pain Threshold | B_R_SbCG_S | 0.09 | 0.9283 | 62 | 21 |
