# Supplementary material for: Evidence for an association of gut microbial Clostridia with brain functional connectivity and gastrointestinal sensorimotor function in patients with irritable bowel syndrome, based on tripartite network analysis
Source: Microbiome. 2019 Mar 21;7:45. doi: 10.1186/s40168-019-0656-z (PMC6429755; doi:10.1186/s40168-019-0656-z)

Supplemental Materials.

Using G*Power, we perform a post hoc analysis to determine the effect size, r, our samples could detect with adequate power (80%) based on an alpha=.05 (uncorrected p), and a two tailed test for a significant correlation. For the IBS sample of 65 subjects, we only had adequate power to detect a significant correlation of r>=.33, if it existed. For the HC sample (N=21) we only had adequate power for r>= .54.

Using this same, model we plot the sample size needed to detect a specific effect size, r, using uncorrected p values (See **Figure 1**).

Figure 1. Effect sizes detectable with adequate power at p<.05 uncorrected as a function of sample size.
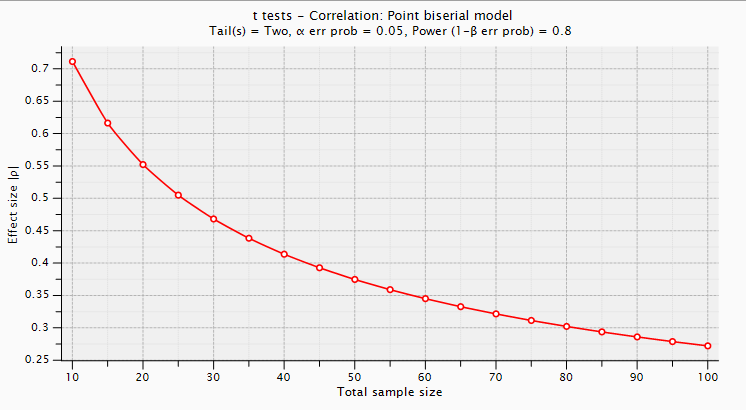


Based on correlating 9 clinical variable, 9 genera, 40 brain regions and 3 types of network metrics, 8640 total tests were performed. The plot in **Figure 2** depicts the sample size necessary to have adequate power to detect effect sizes, ranging from r=.20 to .80 and implementing a Bonferroni correction for multiple tests, a= .05/8640= 0.00000579. In order to provide adequate power and justify tighter error control we would need about 450 subjects to detect an effect of r>=.25, if it exists.

Figure 2. Effect sizes detectable with adequate power at p= 0.00000579 as a function of sample size.


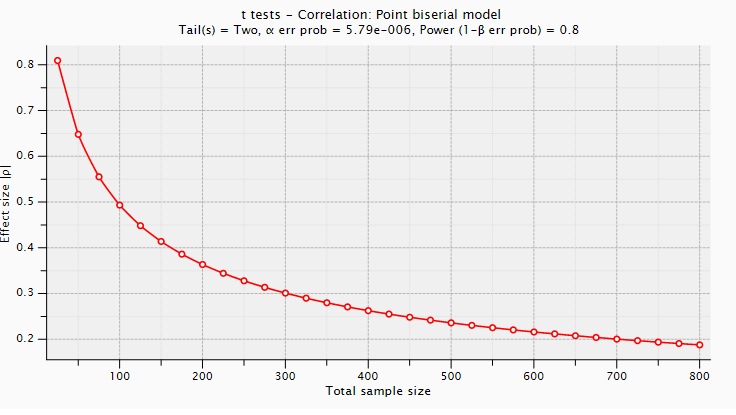

Supplement: Supplementary file 4 — Supplemental Materials. Supplemental materials related to statistical power calculations. (DOCX 71 kb) [file 40168_2019_656_MOESM4_ESM.docx]
